# Single‐cell mitochondrial sequencing reveals low‐frequency mitochondrial mutations in naturally aging mice

**DOI:** 10.1111/acel.14242

**Published:** 2024-06-21

**Authors:** Fuyan Liu, Xiaolin Sun, Cai Wei, Liu Ji, Yali Song, Chenlu Yang, Yue Wang, Xin Liu, Daqing Wang, Jingmin Kang

**Affiliations:** ^1^ BGI Research Beijing China; ^2^ Dalian Maternal and Child Health Hospital of Liaoning Province Dalian Liaoning China; ^3^ State Key Laboratory of Quality Research in Chinese Medicine and Institute of Chinese Medical Sciences University of Macau Macao China; ^4^ BGI Research Shenzhen China

**Keywords:** aging, ATAC, mitochondrial DNA (mtDNA), mitochondrial mutation, single cell sequencing

## Abstract

Mitochondria play a crucial role in numerous biological processes; however, limited methods and research have focused on revealing mitochondrial heterogeneity at the single‐cell level. In this study, we optimized the DNBelab C4 single‐cell ATAC (assay for transposase‐accessible chromatin) sequencing workflow for single‐cell mitochondrial sequencing (C4_mtscATAC‐seq). We validated the effectiveness of our C4_mtscATAC‐seq protocol by sequencing the HEK‐293T cell line with two biological replicates, successfully capturing both mitochondrial content (~68% of total sequencing data) and open chromatin status simultaneously. Subsequently, we applied C4_mtscATAC‐seq to investigate two mouse tissues, spleen and bone marrow, obtained from two mice aged 2 months and two mice aged 23 months. Our findings revealed higher mitochondrial DNA (mtDNA) content in young tissues compared to more variable mitochondrial content in aged tissues, consistent with higher activity scores of nuclear genes associated with mitochondrial replication and transcription in young tissues. We detected a total of 22, 15, and 21 mtDNA mutations in the young spleen, aged spleen, and bone marrow, respectively, with most variant allele frequencies (VAF) below 1%. Moreover, we observed a higher number of mtDNA mutations with higher VAF in aged tissues compared to young tissues. Importantly, we identified three mtDNA variations (m.9821A>T, m.15219T>C, and m.15984C>T) with the highest VAF in both aged spleen and aged bone marrow. By comparing cells with and without these mtDNA variations, we analyzed differential open chromatin status to identify potential genes associated with these mtDNA variations, including transcription factors such as *KLF15* and *NRF1*. Our study presents an alternative single‐cell mitochondrial sequencing method and provides crude insights into age‐related single‐cell mitochondrial variations.

## Graphical abstract

We focused on optimizing the single‐cell ATAC sequencing workflow using the DNBelab C4 platform for mitochondrial analysis. Validating the method on the HEK‐293T cell line, we successfully captured mitochondrial content and simultaneous information on open chromatin status. We also applied it to aged spleen and bone marrow tissues from mice, proved its efficiency in profiling mtDNA variations in tissues.

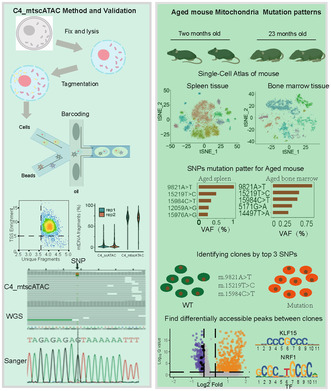

**Abbreviations**
3T‐3‐L1: Mouse Adipocyte‐Like Cell Line1
ATAC: Assay for Transposase‐Accessible Chromatin
Bcl11b: B‐Cell CLL/Lymphoma 11B
CD4+: CD4 antigen positive
CD8+: CD8 antigen negative
EGR3: Early Growth Response 3
HEK‐293: Human Embryonic Kidney 293 Cells
KLF15: Kidney‐Enriched Krueppel‐Like Factor 15
NRF1: Nuclear Respiratory Factor 1
Pax5: Paired Box 5
S1008a: S100 calcium binding protein A8
SP3: Sp9 Transcription Factor 3
SP9: Sp9 Transcription Factor 9

## 1 INTRODUCTION

The advent of microfluidic systems has revolutionized cell handling by enabling the efficient separation of single cells into droplets and the addition of barcodes to each cell (Svensson et al., 2018). This breakthrough has facilitated large‐scale single‐cell sequencing, as microfluidic‐based methods can handle cells on a high‐throughput scale (Luecken & Theis, 2019). Compared to plate‐based methods, microfluidics offers enhanced effectiveness in profiling transcriptomes and chromatin accessibility comprehensively. DNBelab C4 is a portable and cost‐effective microfluidic system specifically designed for single‐cell handling (Liu, Liu, et al., 2019). Previous studies have successfully developed workflows for single‐cell transcriptome sequencing and assaying chromatin accessibility (ATAC) sequencing using DNBelab C4. These workflows have been applied to investigate single‐cell transcriptomes (Han et al., 2021; Liu, Liu, et al., 2019; Zhu et al., 2020) and assess open chromatin status (Lei et al., 2022; Liu, Liu, et al., 2019; Liu, Wang, et al., 2019). While the challenge of massively parallel single‐cell genome sequencing persists across microfluidic‐based methods, it is now feasible to recover released DNA within single cells by incorporating barcoding techniques prior to sequencing, similar to assaying transposase‐accessible chromatin. Notably, microfluidic‐based methods have made it possible to profile mitochondrial DNA (mtDNA) in single cells (Lareau et al., 2021).

Mitochondria are multifunctional organelles that play crucial roles in cells. During the aging process, mitochondrial function declines, accompanied by weakened oxidative phosphorylation activity, changes in levels of mitochondrial tricarboxylic acid cycle enzymes, accumulation of mtDNA mutations, increased reactive oxygen species production, and imbalanced mitochondrial protein homeostasis (Zhu et al., 2022). Therefore, mitochondrial dysfunction may disrupt communications between mitochondria and nucleus, leading to aging‐related gene expression changes and the occurrence of age‐related phenotype or diseases (Moro, 2019). While the human mitochondrial genome (mtDNA) only encodes 13 protein genes, 22 tRNAs, two rRNAs, and a D‐loop regulatory region (Stewart & Chinnery, 2021), there are 1000–1500 proteins in mitochondria and are encoded by most of the nuclear genes (Song et al., 2021). This suggested that the homeostasis within mitochondria may be regulated by nuclear transcription factors and their associated cofactors (Kummer & Ban, 2021).

Both bulk and single‐cell ATAC sequencing data could potentially contain a small proportion of mitochondrial DNA (mtDNA) due to the inclusion of mitochondria and the release of mtDNA, despite nuclei typically being the focus of extraction and analysis. Experimental workflows have been developed to isolate and sequence individual mitochondria, while bioinformatic methods have been devised to extract and analyze mtDNA data from single‐cell ATAC sequencing (Ludwig et al., 2019; Xu et al., 2019). However, mtDNA sequencing data may originate from mitochondria of different cells without specific enrichment of mtDNA in each cell, and the sequencing depth may be insufficient to capture mtDNA variations within individual cells. This is due to the high copy numbers of mtDNA (Calloway et al., 2000; Chomyn & Attardi, 2003; Coxhead et al., 2016; Wallace, 2010). Each cell harbors several hundred copies of mtDNA, where both wild‐type and mutant mtDNAs can coexist (Jayaprakash et al., 2015; Nekhaeva et al., 2002; Yao et al., 2007). Bulk sequencing of heterogeneous cell populations with varying mtDNA compositions may obscure mtDNA variations in individual cells, necessitating the exploration of mtDNA heteroplasmy (the proportion of mitochondria with variable allele at some loci) at the single‐cell level to precisely elucidate the distribution of mtDNA variations within a specific cell population (Guo et al., 2023). Recently, a modified protocol based on the 10× Genomics platform, derived from the single‐cell ATAC protocol, has been reported. This protocol enables the enrichment and sequencing of mtDNA in single cells, demonstrating the feasibility of mtDNA sequencing at the single‐cell level (Lareau et al., 2023, 2021). Despite various efforts to develop single‐cell assays for transposase‐accessible chromatin sequencing (scATAC‐seq) to identify mtDNA mutations and quantify mitochondrial content, there are still areas that require improvement, such as enhancing mtDNA coverage and increasing sequencing depth.

In this study, we have developed a novel protocol, C4_mtscATAC‐seq, for single‐cell mtDNA sequencing using the DNBelab C4 platform. To demonstrate its applicability, we conducted a proof‐of‐concept investigation into the heterogeneity of mitochondrial content and mutations at the single‐cell level in aging mice. We identified three mutations (m.9821A>T, m.15219T>C, and m.15984C>T) present in both aged spleens and bone marrows. Notably, we observed distinct chromatin accessibility profiles between cells carrying these mitochondrial mutations and cells lacking these mutations. Furthermore, we identified potential associations between these mutations and specific transcription factors, such as *KLF15* and *NRF1*. Our study sheds light on the mitochondrial heterogeneity associated with aging, leveraging the newly established C4_mtscATAC‐seq protocol.

## 2 RESULTS

### 2.1 Establishing and optimizing the C4_mtscATAC‐seq protocol

**FIGURE 1:**
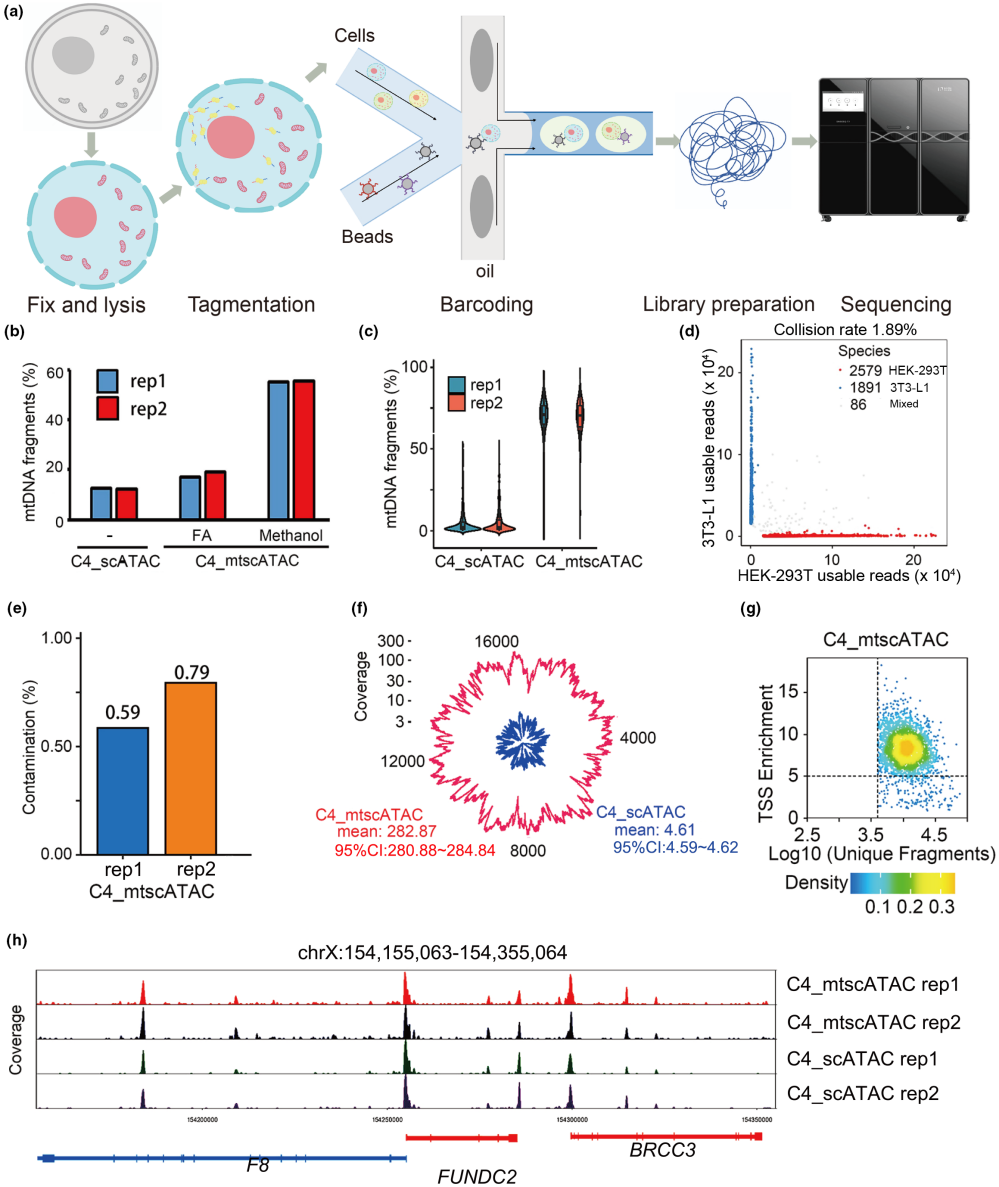
Establishing the C4_mtscATAC‐seq workflow and proving its efficiency through sequencing HEK‐293 T cell line. (a) Schematic of C4_mtscATAC‐seq workflow. (b) Mitochondrial DNA sequence proportions resulted from using different fixation reagents. Fixation reagents of formaldehyde (FA) and methanol were test in C4_mtscATAC. Each experiment included two biological replicates. (c) Mitochondrial DNA sequence proportions of C4_scATAC‐seq and C4_mtscATAC‐seq experiments. (d) Collison estimation of C4_mtscATAC‐seq. Successful droplets with sequencing reads uniquely mapped to either human or mouse genome were categorized as singlets. Otherwise, they will be categorized as mixed (*n* = 86). (e) Contamination estimation of C4_mtscATAC‐seq. (f) Mitochondrial genome coverage by C4_mtscATAC‐seq data compared to C4_scATAC‐seq data. The numbers next to the graph represent the mean coverage depth and the corresponding 95% confidence interval (CI) value. In panels c and f, each condition represents the top 1000 cells (based on chromatin complexity) for each replicate. (g) Transcription starting sites (TSS) enrichment of C4_mtscATAC‐seq data. The scatter plot represents the number of unique ATAC‐seq nuclear fragments in each cell, along with the TSS enrichment of all fragments within that cell. The dashed lines indicate the thresholds for high‐quality single‐cell data, requiring a minimum of 4000 unique nuclear fragments and a TSS score equal to or greater than 5. The density on the graph is presented in arbitrary units. (h) Examples of open chromatin profiling using different methods. The accessible chromatin landscapes aggregated from single cells located in the chrX:154,155,063‐154,355,064 region was shown in both regular C4_scATAC‐seq and C4_mtscATAC‐seq data.

DNBelab C4 is a microfluidic system designed for cost‐effective and portable single‐cell sequencing. In our initial workflow, we employed formaldehyde cross‐linking, following the mtscATAC‐Seq protocol developed for the 10x Genomics single‐cell platform (Lareau et al., 2021), to immobilize mitochondria within the cells. Subsequently, we utilized the C4 DNBelab single‐cell platform for downstream scATAC‐Seq library preparation. However, we observed that the polymerase might encounter difficulties in traversing protein‐crosslinked DNA regions during the PCR reaction after generating single‐cell Gel Bead‐In‐Emulsions on the C4 DNBelab platform, resulting in suboptimal DNA capture from nuclear open chromatin regions. To address this issue, we modified our C4_mtscATAC‐seq protocol by adopting a gentler cross‐linking agent, cold methanol, to fix the cells. This adjustment improved the fixation efficiency on the DNBelab C4 platform. We tested our method in cell lines and chose a mixture of human HEK‐293 T cells and mouse 3T3‐L1 cells with two biological replicates for sequencing using our C4_mtscATAC‐seq protocol (Figure 1a). Using the formaldehyde fixation, we only obtained approximately 19.42% and 11.09% of mtDNA fragments in HEK‐293T and 3T3‐L1 cells, respectively (Figure 1b and Figure S1a). Using the cold methanol (MeOH), we successfully improved the percentages of mtDNA fragments up to approximately 67.79% in HEK‐293T (Figure 1c) and 60.01% in 3T3‐L1 (Figure S1b). To unveil the relationship between mitochondrial content and copy number, we utilized qPCR to accurately estimate the actual copy number. The findings indicated that the copy number in HEK‐293T cells was significantly higher than that in 3T3‐L1 cells (Figure S2 and Table S1). This observation suggests a consistent trend with the results of mitochondrial content, which provide a rough estimate of copy numbers for each cell, facilitating comparisons between samples or cell types. Furthermore, we found the enrichment at transcription start sites (TSS) to be improved compared to formaldehyde fixation (Figure S3).

We then evaluated the performance of the optimized C4_mtscATAC‐seq protocol in single‐cell profiling. Firstly, we examined the distribution of the percentage of mtDNA fragments per single cell and observed that the mitochondrial content derived from C4_mtscATAC‐seq was higher than C4_scATAC‐seq (Figure 1c and Figure S1b). We analyzed the collision rate, reflected by single‐cell data containing both human and mouse sequences, to be approximately 1.89% (Figure 1d). Furthermore, we assessed the contamination, where single‐cell data contained both human and mouse mtDNA sequences, indicating the presence of mitochondria from more than one cell in the single‐cell data. The contamination rate was estimated to be lower than 1% (Figure 1e).

Overall, our C4_mtscATAC‐seq using cold methanol for fixation, leading to a ~ 61.36‐fold (from 4.61× to 282.87×) and 61.84‐fold (from 2.04× to 126.16×) increase in mean mtDNA coverage per cell comparing to C4_scATAC‐seq under similar total sequencing amount in a separate HEK‐293T and 3T3‐L1 cell line, respectively (Figure 1f and Figure S1c). Meanwhile, the data of the established C4_mtscATAC‐seq can also be used for analyzing open chromatin (Figure 1g and Figure S1d), with about 25.64% in and 33.85% of Tn5 insertion sites to be within peaks present in all aggregated cells of HEK‐293T and 3T3‐L1, respectively (Table S1), indicating that the optimized C4_mtscATAC‐seq can effectively obtain both nuclear open chromatin DNA and mtDNA simultaneously. We identified chromatin peaks using aggregate C4_mtscATAC‐seq profiles, which showed a strong correlation with peaks identified using C4_scATAC‐seq data (Pearson correlation = 0.88). Examples of peaks related to genes (*FUNDC2*, *F8*, and *BRCC3*) are shown in Figure 1h.

### 2.2 Profiling single‐cell mtDNA variability in HEK‐293T cells

**FIGURE 2:**
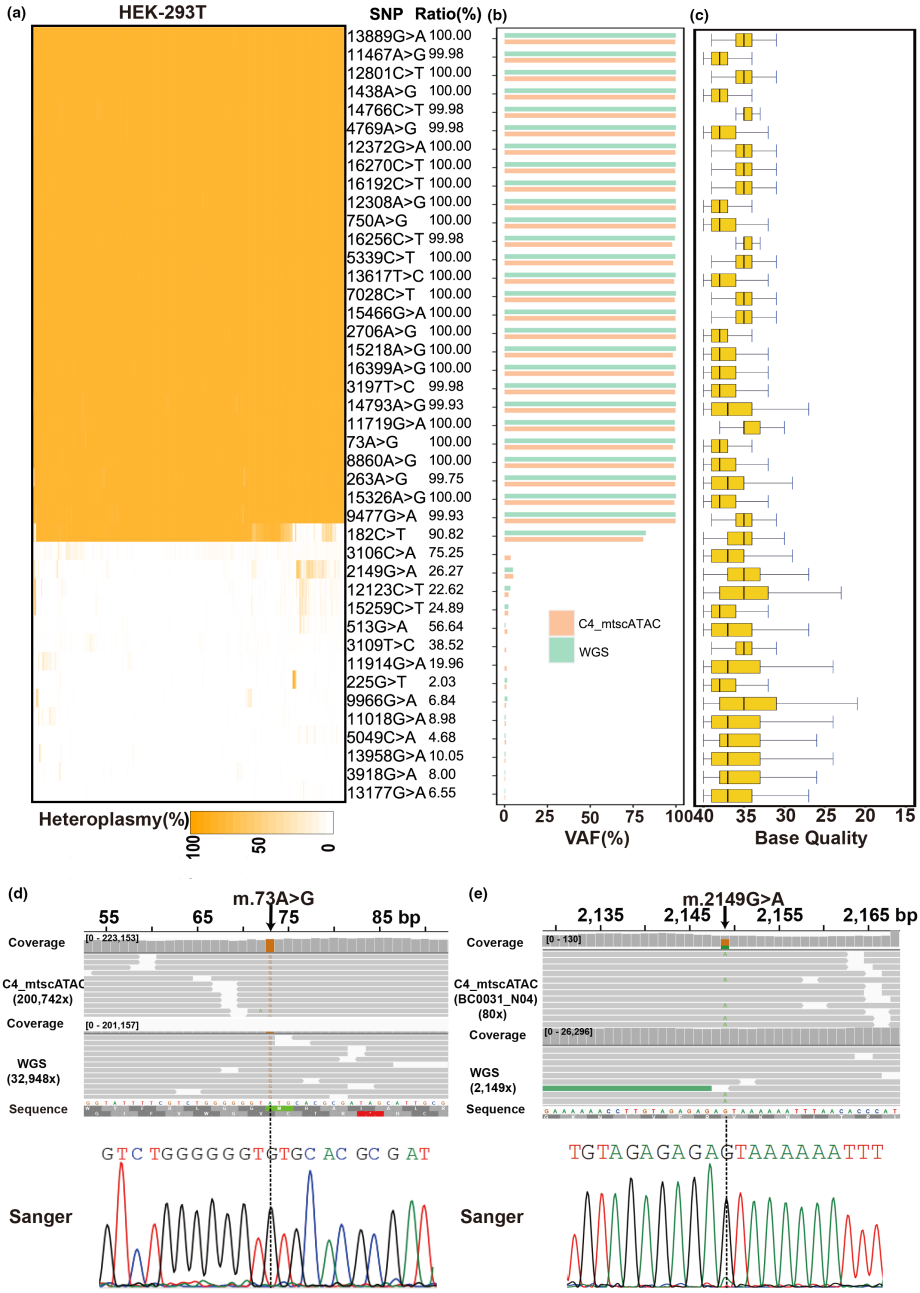
Depicting mtDNA variations in HEK‐293 T cell using C4_mtscATAC‐seq. (a) 42 high‐quality variations identified and the three groups of cells. Each column represents an individual cell, and the rows indicated the identified variations. The heatmap color indicates the percentage of heteroplasmy. The ratio of cells harboring corresponding SNPs were also be listed alongside. (b) Variant allele fraction (VAF) frequency of each mutation calculated based randomly selected 1000× WGS data (cyan bars) and C4_mtscATAC‐seq data (orange bars). (c) The distribution of base quality scores from the C4_mtscATAC‐seq sequencing reads supporting each mutation. (d and e) Visualized the alignments of WGS and C4_mtscATAC‐seq reads near m.73A > G and m.2149G > A (used one cell data) by the Integrative Genomics Viewer (IGV). The supporting sequencing depth for two locus were listed on the left‐hand side in the IGV viewer and the sanger sequence electropherograms were listed in the below.

We utilized our optimized protocol and the generated data to profile single‐cell mtDNA mutations in HEK‐293T cells, which are derived from human embryonic kidney cells and transformed with the SV40 large T antigen. This case serves as an example to demonstrate the effectiveness of our protocol. We identified a total of 42 mtDNA mutations, among which 28 mutations were also detected using ~110,000 × bulk genome sequencing of the same batch of HEK‐293T cells (Figure 2a, Figure S4 and Appendix S2: SD01). For these 28 mutations, we observed that 27 had a very high population heteroplasmy (>99.9%, Figure 2b and Appendix S2: SD01) by analyzing the C4_mtscATAC‐seq data, indicating that they were specific to this cell line. The remaining mtDNA mutation m.182C>T distributed in 90.82% of cells and had heteroplasmy estimated at 81.13% (Figure 2a), suggesting the presence of cellular heterogeneity and the mtDNA mutations are in relatively high frequency within the cell line. Furthermore, we selected three mitochondrial variations at different frequencies for experiment validation, including m.73A>G (freq. 99.85%), m.2149G>A (freq. 4.80%), and m.13177G>A (freq. 0.24%). For the first two variations at relatively high frequencies, the sanger sequencing results supported them to have variable alleles (Figure 2d,e). But for the last variation with very low frequency, we found the sequencing result did not support it as variations, which may reflect either discrepancies for the very low frequency variations, or detection limitation of the sanger sequencing method. To minimize false‐positive results as much as possible, especially for lower frequency variants with heteroplasmy below 5%, we utilized seven filtering methods (see Section 4) to reduce false positives and prevent the introduction of spurious mutations caused by sequencing errors. All 42 identified variants were distributed in at least 200 cells except for m.225G>T (Appendix S2: SD01) and supported by a minimum of 1000 mapped reads and a base quality of 30 (Figure 2c and Appendix S2: SD01). This indicates the effectiveness of C4_mtscATAC‐seq in depicting low frequency mtDNA variations and revealing the cell heteroplasmy of mtDNA. We further annotated the identified mtDNA mutations to find 13 nonsynonymous and 12 synonymous mutations. We also found nine of these mutations to be presented in kidney cancer samples from TCMA (The Cancer Mitochondrial Atlas) (Yuan et al., 2020), suggesting a potential association with the enhanced proliferation of HEK‐293T cells (Appendix S2: SD02). Collectively, these findings demonstrate the successful optimization of a C4_mtscATAC‐seq based on the DNBelab C4 platform, offering an alternative approach for single‐cell mitochondrial studies.

### 2.3 Analyzing mouse spleen and bone marrow using C4_mtscATAC‐seq

The optimized C4_mtscATAC‐seq method demonstrated excellent performance with cell lines, prompting us to extend its application to mouse tissues. We applied C4_mtscATAC‐seq in spleens from two young (two‐month‐old) and two aged male mice (23‐month‐old) as well as in the bone marrow of aged male mice. While acknowledging that two‐month‐old mice may not fully represent adult mice, we employed them for this proof‐of‐concept comparison to document mtDNA variations in aged mouse tissues. For each of these three tissue samples, we constructed four C4_mtscATAC‐seq libraries. We obtained 10,702, 11,654, and 14,088 single cells for these three tissue samples, respectively. We found the average mitochondrial content (~51.52%), number of unique fragments mapped to the nuclear genome per cell (~6412.63), as well as the percentage of the nuclear fragments to be overlap with peaks (~37.94%), all supported success profiling of both the mitochondria and the open chromatin in single cells (Figure S5a,b and Table S2).

To confirm the reproducibility of our experiments, we performed a correlation analysis of the aggregate peak profiles and mitochondrial sequencing depth among three tissue samples. This analysis revealed high correlations (average Pearson correlation coefficient >0.87) between different replicates (Figure S6), again validating the reproducibility of our experimental approach. Additionally, upon visual examination of the aggregated spleen C4_mtscATAC‐seq profiling data, we observed that the open chromatin peaks were consistent with previously published scATAC data (Mogilenko et al., 2021) (Figure S5c). To delve deeper into the analysis, we examined marker genes associated with the coefficient of spleen aging. These marker genes were derived from a previously published single‐cell RNA sequencing (scRNA‐seq) and scATAC study (Chen et al., 2018; Giladi et al., 2018; Lareau et al., 2021). Our analysis demonstrated that the patterns of these marker gene activity scores in our young and aged C4_mtscATAC‐seq spleen samples aligned with scRNA‐seq data (Tabula Muris, 2020) (Figure S7a). This agreement provides further evidence of the quality and reliability of our C4_mtscATAC‐seq method in exploring the mitochondrial heterogeneity of mouse tissues.

**FIGURE 3:**
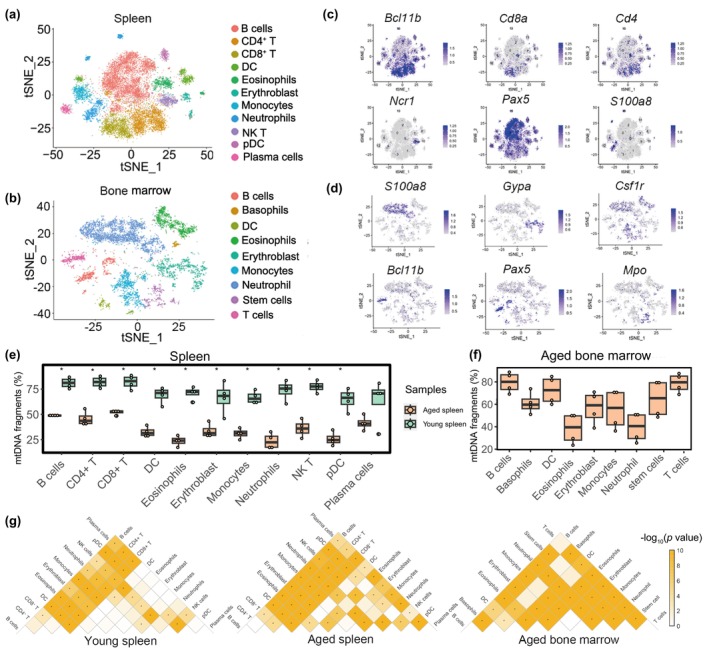
Cell type annotation and mitochondrial contents profiling in mouse tissues according to C4_mtscATAC‐seq data. (a and b) t‐Distributed Stochastic Neighbor Embedding (t‐SNE) projection of mouse spleen cells (a) and bone marrow cells (b) based on the C4_mtscATAC‐seq data. (c and d) The same t‐SNE plot as shown in panels a and b, with colors indicating the gene activity score of the indicated genes. (e) The mtDNA content in each cell type from C4_mtscATAC‐seq data of young and aged spleens. This estimation was performed by calculating the median proportion of fragments mapped to the mitochondrial genome in each cell type for each sample. Significance was determined using the Wilcoxon test, where * represents *p* < 0.05. (f) The median mtDNA content for each cell type in aged bone marrows. (g) Heatmap illustrating the significance of differences in mtDNA content among different cell types in young spleen, aged spleen and aged bone marrow. Cells displaying a *p*‐value <0.01 in the Wilcoxon test are denoted with an asterisk (*). Orange indicates higher significance, while white indicates lower significance.

Based on the obtained open chromatin information, we performed cell cluster annotation and identified 11 major cell types in the spleen and nine major cell types in the bone marrow (Figure 3a,b and Table S3). Cluster identities were defined using gene activity scores. For instance, the *Bcl11b* gene score marked T cells, the *Pax5* gene score marked B cells, and the *S1008a* gene score marked neutrophil cells (Figure 3c,d and Table S4). Overall, we successfully applied the C4_mtscATAC‐seq method to explore mitochondrial heterogeneity across diverse cell types in mouse tissues.

### 2.4 Mitochondrial DNA content heterogeneity between different cell types

Mitochondrial DNA copy number (mtDNA‐CN) is a reliable indicator of mitochondrial function and has been associated with human diseases (Castellani et al., 2020; Hosnijeh et al., 2014; Mengel‐From et al., 2014). In our study, we estimated the relative quantity of mtDNA‐CN by mtDNA content. This was calculated as the percentage of sequencing fragments that were mapped to mtDNA (Tang et al., 2022). To investigate changes in mtDNA‐CN during spleen aging in two mice, we examined various cell types (Figure 3e). We found mtDNA content in B cells, CD4^+^ T cells, and CD8^+^ T cells to be significantly higher compared to other cell types in both young and aged mouse spleens (Figure 3g). More importantly, mtDNA content was significantly higher in young spleens compared to aged spleens across all cell types except for plasma cells (Figure 3e). Previous research has demonstrated a strong association between the expression levels of genes involved in mtDNA replication and transcription and actual mtDNA‐CN (Campbell et al., 2012; Ekstrand et al., 2004; Tang et al., 2022; Yang et al., 2021). To support the accuracy of our estimated mtDNA‐CN from C4_mtscATAC‐seq data, we computed mtDNA‐CN‐related gene signature scores for each cell. Remarkably, we found significantly higher scores in most cell types derived from young spleens compared to their counterparts in aged spleens (Figure S7b and Table S5), supporting the use of the percentage of mtDNA as an indirect measure for quantifying mtDNA in our C4_mtscATAC‐seq data. Next, we analyzed mtDNA content in samples of aged bone marrow tissue. Our findings revealed that B cells and T cells exhibited the highest levels of mitochondrial content, while neutrophils showed the lowest levels (Figure 3f), consistent with observations in aged spleen tissue. Finally, we calculated the differences in mtDNA content between different cell types in young spleens, aged spleens, and bone marrow samples, respectively. We observed an amplified heterogeneity among cell types during the spleen aging process, with the number of significant pairs increasing from 34 to 45 (Figure 3g). For instance, in the aged spleen sample, the mtDNA content of plasma cells showed significant differences compared to all other cell types, whereas in the young spleen sample, it only differed significantly from B cells, CD4^+^ T cells, and CD8^+^ T cells (Figure 3g). In contrast, no significant differences were found between spleen and bone marrow tissues during the aging process (45 pairs accounted for 0.82 out of a total of 55 groups in the aged spleen and 0.83 (30/36) in the aged bone marrow). Thus, we illustrated the mitochondrial DNA content heterogeneity to be more variable along aging in the two mouse tissues.

### 2.5 mtDNA variations in spleens and bone marrows

**FIGURE 4:**
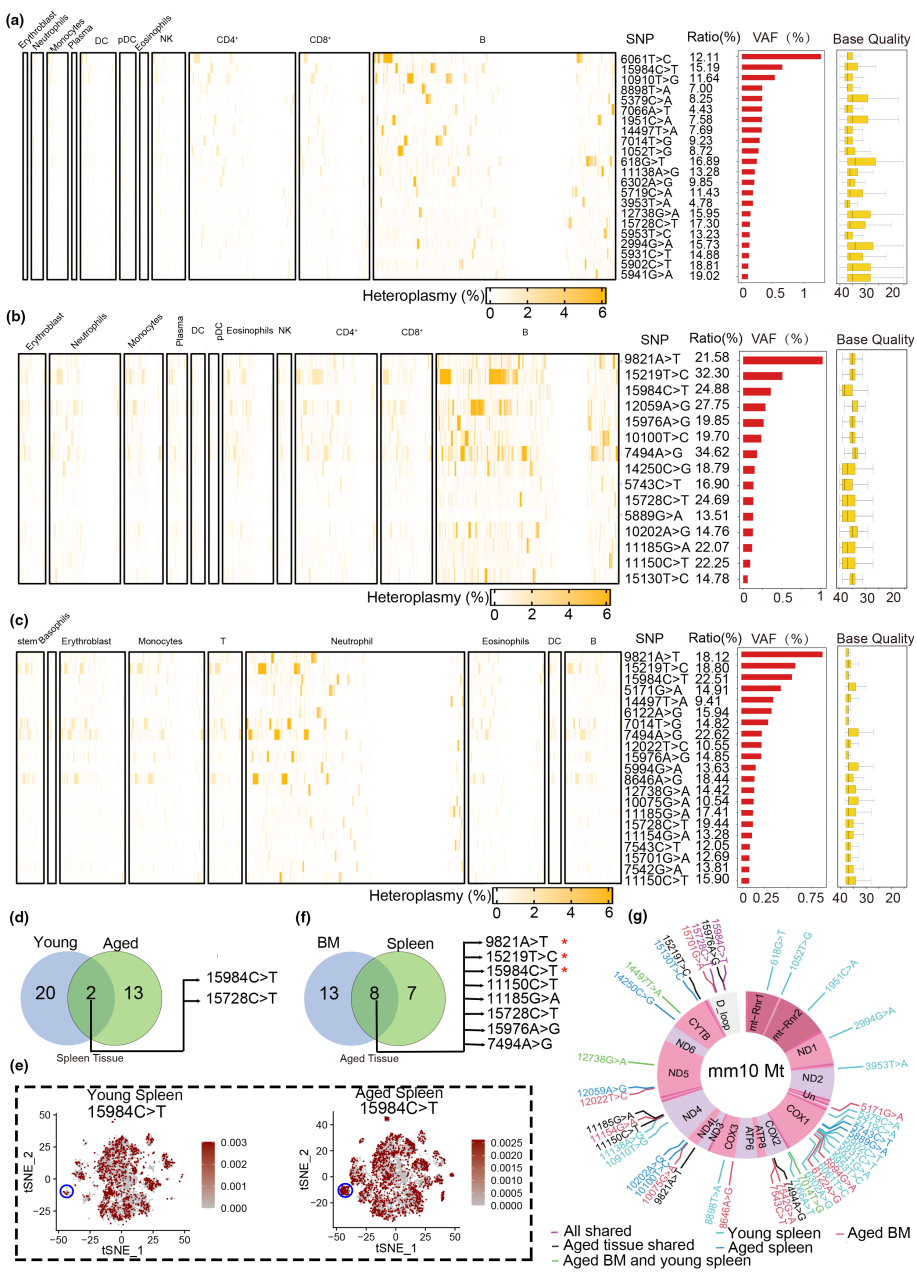
The single‐cell mtDNA variations in mouse tissues revealed by C4_mtscATAC‐seq. (a) Heatmap displaying the allele frequency of 22 informative mtDNA variations detected in each cell type of the young spleen. The ratio of mutated cell, variant allele frequency (VAF) based on pseudo‐bulk data and base quality distribution are listed on the right side. (b, c) 15 mtDNA variations identified in aged spleen (b), and 21 mtDNA variations were identified in aged bone marrow (c). (d) Venn diagram illustrating the count of overlapping variations between young and aged spleens. Two variations, m.15984C > T and m.15728C > T, were found to be shared between young and aged spleens and m.15984C > T was also observed between aged tissues (see f). (e) Visualization of allele frequencies of the m.15984C > T variation across cells. A specific cell type (plasma cells) has been circled in blue, demonstrating the expansion of the variation in aged spleens. (f) Venn diagram illustrating the count of overlapping mutations between aged spleen and aged bone marrow. Eight shared mutations are listed on the right, and the top three mutations with the highest VAF in each tissue were marked with a red asterisk. (g) Landscape of mtDNA mutations in three groups (young spleen, aged spleen, and aged bone marrow). A circular plot of the mitochondrial genome with annotated gene locations is displayed. Each mutation is labeled based on its coordinates on the mitochondrial genome, and different colors indicate consistency among the three groups.

Most heteroplasmies in mice were observed at very low variant allele frequencies (VAFs) of 1%–2% or lower (Arbeithuber et al., 2020; Sanchez‐Contreras et al., 2023). Conventional bulk sequencing methods have limitations in detecting these low‐frequency heteroplasmic variations due to lower coverage of mitochondrial DNA and the inability to detect them at the single‐cell level. Thus we identified mtDNA variations in single cells using the generated C4_mtscATAC‐seq data. To ensure the accuracy of variant identification, we implemented multiple filtering methods, as described in the Section 4. Through this process, we identified a total of 22, 15, and 21 mtDNA variations in the young spleen, aged spleen, and aged bone marrow, respectively, from each of two mice (Figure 4a–c, and Appendix S2: SD03). Statistical analysis of aggregated single‐cell data demonstrated that most mutations had variable allele frequencies (VAFs) as low as 1% at the bulk level, indicating that C4_mtscATAC‐seq enabled the detection of low‐frequency mutations. Analysis of the mutation distribution did not reveal any cell type‐specific mutations. We further annotated the identified mutations and found that 77.27% of mtDNA variations in young spleen, 73.33% in aged spleens, and 71.43% in aged bone marrow were in protein‐coding genes (Appendix S2: SD04). Among these variants, 81.25%, 72.73%, and 78.57% were nonsynonymous (Figure S8a). Notably, mtDNA variations affected genes such as tRNA‐Arg, tRNA‐Val, *COX1*, *COX2*, *COX3*, *CYTB*, *ND1*, *ND2*, *ND4*, *ND4L*, and *ND5* (Figure S8a). When comparing the two tissues from the aged mice, we observed a similar pattern of mutations in these genes, with more variations detected in *COX2* and tRNA‐Val in the bone marrow cells.

We then conducted a comparison of variation differences among the three samples (young spleen, aged spleen, and aged bone marrow). We identified two shared mtDNA variations, m.15984C>T and m.15728C>T, between the young and aged spleen tissues (Figure 4d). The m.15984C>T mutation was present in 15.19% of young spleen cells and 24.88% of aged spleen cells. Similarly, the m.15728C>T mutation was observed in 17.30% of young spleen cells and 24.69% of aged spleen cells (Appendix S2: SD03). Taking m.15984C>T as an example, we observed an increased number of mutated cells in the aged spleen (evident in plasma cells, as indicated by the arrows in Figure 4e). These findings suggest that mtDNA variations are more prevalent in aged cells of spleen. Furthermore, we examined the differences between the aged tissues and identified eight shared variants (Figure 4f), among which three mtDNA mutations, m.9821A>T, m.15219T>C, and m.15984C>T, had the highest VAFs at pseudo‐bulk level (Figure 4b,c). These intriguing results provide evidence for a potential association between these three specific mtDNA variations and the aging process. The landscape of all the identified variations in the mtDNA were visualized in Figure 4g. We also observed that one of the shared mtDNA variations in the aged tissues, m.11185G>A, located in NADH–ubiquinone oxidoreductase chain 4 (*ND4*), resulted in an amino acid change from Arginine (R) to Histidine (H). Notably, this variation is homologous to m.11778G>A in humans (Figure S8b–d), which has been previously reported to be associated with Leber hereditary optic neuropathy (LHON) (Newman et al., 2023). This suggests that m.11185G>A may play a role in certain aging processes.

### 2.6 Cell type‐specific mtDNA variation patterns

**FIGURE 5:**
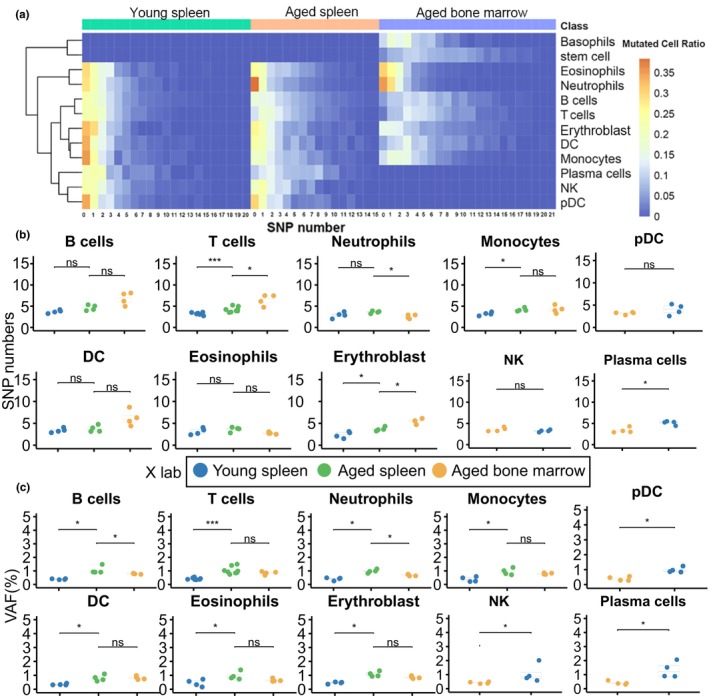
Cell‐type specific mtDNA variations in mouse spleen and bone marrow. (a) Heatmap illustrating the percentages of cells carrying different numbers of mtDNA variations in young spleens, aged spleens, and bone marrows. The color scale ranges from red, indicating a higher proportion of mutated cells, to blue, indicating a lower proportion. (b) Comparison of the average number of mutations carried by each cell type between young spleen and aged spleen, as well as between aged spleen and aged bone marrow. The samples information were shown in the legend. (c) Comparison of the average VAF of mtDNA variations of each cell type between young spleen and aged spleen, as well as between aged spleen and aged bone marrow. The samples information were the same as b. Significance was determined using the Wilcoxon test. Statistical significance is denoted as follows: * for *p* < 0.05, ** for *p* < 0.01, *** for *p* < 0.001.

We conducted a statistical analysis to examine the number of mutations in each cell type and observed that most cells harbored three or fewer mutations. Notably, there was a moderate increase in the number of mutations in two aging tissues (Figure 5a). Among the analyzed cell types, four cell types (T cells, monocytes cells, erythroblast, and plasma cells) exhibited significantly higher mutation numbers in aged spleens compared to young spleens, while the remaining four cell types did not show a significant difference (Figure 5b). We focused specifically on neutrophil cells in aged tissues and observed a higher proportion of cells without mutations (represented by values over 0.35 in Figure 5a) compared to other cell types. This suggests that neutrophils may play a significant role in combating the aging process. However, it is important to note that an increased number of mtDNA variations in immune cells could potentially impact their immune activity, potentially leading to reduced immunity during aging (Beadnell et al., 2020; Schilf et al., 2021). Furthermore, we performed a further analysis comparing aged spleen and bone marrow. We found that T cells and erythroblast exhibited significantly higher variation numbers in the aged bone marrow compared to the spleen (Figure 5b). Conversely, neutrophils showed significantly higher variation numbers in the spleen compared to the bone marrow (Figure 5b). These results suggest the potential for tissue‐specific and cell type‐specific patterns for mtDNA mutations.

Subsequently, we performed an analysis of the VAF of mtDNA variations at the single‐cell level. Consistently, we observed that all cell types exhibited higher VAF in aged spleens compared to young spleens (Figure 5c). Additionally, we conducted a comparison between aged spleens and aged bone marrow. We discovered that B cells and neutrophils had higher VAF in the aged spleen compared to the aged bone marrow, while other cell types exhibited similar VAF between the two aged tissues. These observed variations in mtDNA variation likely reflect changes in cellular function, although further investigations are needed to fully understand their significance.

### 2.7 Possible association between open chromatin and mtDNA variations

**FIGURE 6:**
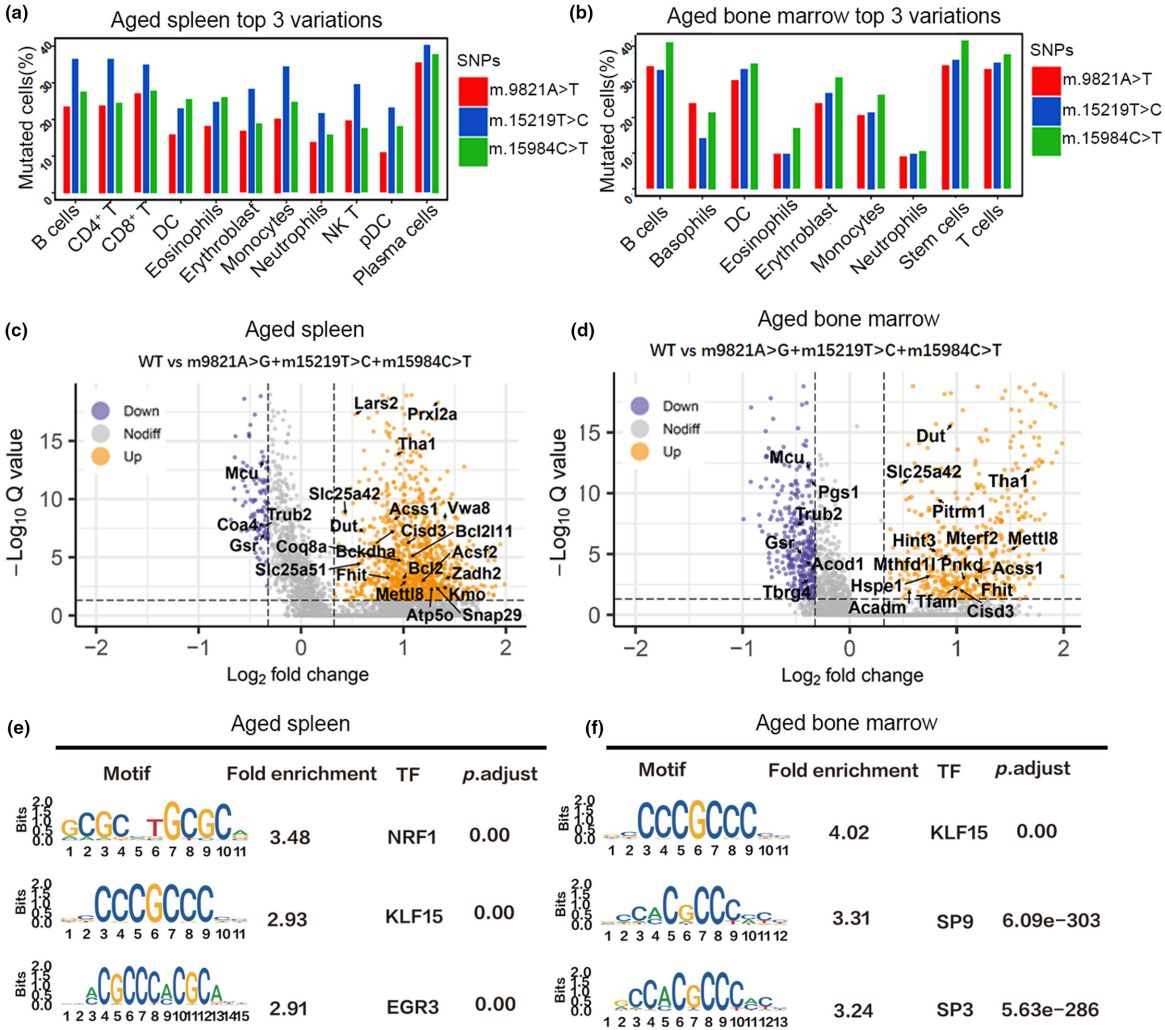
Most widely exhibited mtDNA variations and their possibly associated open chromatin status. (a and b) Barplot displaying the percentages of cells carrying the m.9821A > G, m.15219 T > C, and m.15984C > T mutations in each cell type of the aged mice spleens (a) and bone marrows (b). (c, d) Volcano plot illustrating differential open peaks between the wild‐type (WT) and mutation groups in aged spleen (c) and bone marrow (d), respectively. Cells from aged mice spleens without the three variations (m.9821A > G, m.15219 T > C, and m.15984C > T) were assigned to the WT group, while the remaining cells were assigned to the mutations group. Vertical dashed lines represent the log_2_(Fold change) cut off of 1.25‐fold, while the horizontal line refers to the Q value cut off of Q = 0.01 expressed as −log (Q value).The differential peaks associated with mitochondria‐associated genes are labeled. (e, f) The top three over‐represented transcription factor motifs identified from the differential peaks between the two groups.

Emerging evidence suggests that chromatin modifications play a critical role in mediating communication between the mitochondria and the nucleus in response to mitochondrial disturbances (Burr et al., 2023; Hosnijeh et al., 2014; Minocherhomji et al., 2012; Zhu et al., 2022). In instances where mitochondrial function is compromised, such as through the accumulation of mtDNA mutations, alternative pathways are activated to mitigate the damage and maintain cellular homeostasis (Gonzalez‐Freire et al., 2015). In line with this, our study identified three shared mtDNA variants (m.9821A>T, m.15219T>C, and m.15984C>T) with the highest mutation frequencies in both aged spleen and bone marrow (Figure 4f). Among these variants, m.15219T>C, located in *CYTB* gene (exon1:c.T1075C:p.F359L), exhibited the highest abundance across all cell types in the aged spleen (Figure 6a). In the aged bone marrow, the m.15984C>T mutation, annotated to be in the D‐loop region, was the most prevalent in all cell types except for basophil cells, which carried the highest abundance of the m.9821A>T mutation annotated in tRNA‐Arg (Figure 6b).

Approximately 50.56% of cells in the spleen were found to harbor at least one of the three mutations, while in the bone marrow, this proportion was at least 40.78%. Most cells carried only one mutation, with 21.28% in the spleen and 14.67% in the bone marrow containing two or more mutations. An in‐depth analysis of mutation distribution across different cell types revealed unequal distributions among them (Figure 6a,b). Specifically, mutations were more prevalent in B cells, T cells, plasma cells (spleen), and stem cells (bone marrow). To investigate the impact of mitochondrial mutations on chromatin accessibility in aged spleen and bone marrow, we identified 1069 and 772 differentially accessible peaks (Appendix S2: SD05 and SD06) between cells carrying at least one of the three mutations and cells without any of these mutations (wild type, WT), respectively (*p*‐value adjusted <0.01 and fold change threshold set at 1.25). Furthermore, we randomly divided all cells in the aged spleens into two groups based on the mutation‐to‐WT ratio and calculated the differential peaks between these groups. We repeated this experiment 100 times but did not observe any significant differences in the peaks. Similarly, in aged bone marrow, we conducted a similar analysis and found no significant differential peaks. These findings suggest a correlation between mitochondrial mutations and chromatin accessibility as a possibility.

We conducted further investigations into the differential peaks associated with nuclear genes related to mitochondria. Interestingly, we observed that four nuclear‐mitochondrial genes (*Trub2*, *Gsr*, *Coa4*, and *Mcu*) exhibited decreased chromatin accessibility in the mutation cells of aged spleens, while 20 nuclear‐mitochondrial genes showed increased accessibility, including *Lars2*, *Tha1*, *Prxl2a*, *Bcl2*, and others (Figure 6c). In aged bone marrow, we found that 18 nuclear‐mitochondrial genes displayed increased accessibilities, such as *Tfam*, *Slc25a42*, *Lars2*, *Acss1*, and others, while 6 nuclear‐mitochondrial genes exhibited decreased accessibility, including *Trub2*, *Gsr*, *Mcu*, and *Pgs1* (Figure 6d). To identify potentially specific regulatory sequences associated with mitochondrial variations, we searched for over‐represented DNA motifs in a set of peaks that displayed differential accessibility between the mutation and wild‐type (WT) cells. The top three most significant transcription factor motifs found in aged spleen were *NRF1*, *KLF15*, and *EGR3* (Figure 6e), while in aged bone marrow, the top motifs were *KLF15*, *SP9*, and *SP3* (Figure 6f). Notably, the transcription factor *KLF15*, which has been recently reported to increase mitochondrial cristae and mitochondrial numbers (Gao et al., 2023), was shared by both aged tissues. This suggests that the identified transcription factors in two aged mouse tissues possibly be involved in the response to mtDNA mutations and provide valuable insights into mitochondrial‐to‐nuclear communication.

## 3 DISCUSSION

Single‐cell mitochondrial sequencing provides opportunity for exploring the heterogeneity of mitochondria in individual cells. Although multiple methods developed for studying mitochondrial DNA (mtDNA) at the single‐cell level (Edgar & Myers, 2005; Guo et al., 2023; Lin et al., 2023; Shakir & Li, 2023), they were limited to mitochondrial sequencing thus lacking chromatin accessibility data in one experiment. The recently reported mtscATAC‐seq method based on the 10X Genomics platform effectively addresses this issue and allows for the simultaneous profiling chromatin accessibilities and mtDNA variations within individual cells. However, this method is not compatible with the DNBelab_C4 platform. Therefore, we have developed a cost‐effective single‐cell mitochondria DNA sequencing protocol (C4_mtscATAC‐seq) using the DNBelab_C4 microfluidic system, which is comparable to the mtscATAC‐seq but having more percentage of mtDNA fragments.

Mitochondrial dysfunction is considered a hallmark of aging, accompanied by a decline in oxidative phosphorylation (OXPHOS) function. While investigations of mtDNA mutations in naturally aged mice have been conducted in multiple tissues (Sanchez‐Contreras et al., 2023), comprehensive understanding the mtDNA contents and mutations patterns at single‐cell level remain limited. In this study, we conducted benchmark tests on the HEK‐293T cell line at both bulk and single‐cell levels. The results showed that the high‐frequency mutation sites identified in C4_mtscATAC experiments were all supported by bulk mtDNA data, while 14 low frequency variations were only identified in C4_mtscATAC (Figure S4c). We applied our new protocol to two tissues from young to aged mice (Figure 3), and successfully established the first single‐cell mitochondrial maps in the spleen and bone marrow. Initially, we used mtDNA content to reflect mitochondrial copy number, and found higher mitochondrial content in young spleens than aged spleens (Figure 3), supported by gene activity scores related to mitochondrial replication and transcription. Due to potential bias from technical factors, we believe that using mtDNA content to make comparison of mitochondrial copy numbers among different cell types in the same sample is more reliable than across different tissues. Subsequently, we identified dynamically changing mtDNA variations across different tissues and stages and almost of the variations detected were low frequency (VAF <1%) (Figure 4). Although we utilized seven filtering methods (see Section 4) to mitigate the inclusion of false‐positive mutations caused by sequencing errors, extra validation is required when dealing with these extremely low‐frequency variants. Despite aged cells displaying fewer mtDNA variations overall, we found that the majority of cell types in aged cells had a higher abundance of mtDNA variations and higher value of VAF compared to young cells (Figure 5), indicating a decreased efficiency for clearing mutations during aging (Kong et al., 2022). Moreover, different mutation patterns were observed in different tissues, suggesting tissue‐specific influences of age on mtDNA mutation burden. Finally, we found the specific mtDNA variations (m.9821A>T, m.15219T>C, and m.15984C > T) in aged cells could influence the accessibility of different genes, suggesting mtDNA mutations may serve as signals for mitochondrial‐to‐nuclear communication in aging and potentially resulting in functional changes (Figure 6). Nevertheless, further research is needed to investigate the precise molecular mechanisms of the three variations and its connection to genomic epigenetic changes caused by aging. In summary, through the implementation of our established C4_mtscATAC‐seq protocol, we employed a simple and effective study design to demonstrate its efficiency in capturing chromatin accessibility information and mtDNA sequences and mutations at the single‐cell level. Although mice are our primary research subjects, our studies are valuable for promoting human aging research due to the mice's short lifespan, ease of breeding and rearing, and genetic and physiological similarities to humans (Yuan et al., 2011). By leveraging this protocol, we anticipate the ability to profile mtDNA variations in diverse scenarios about aging and investigate their functional significance within individual cells.

In our investigation, we focused on studying mtDNA variations in young and aged tissues to uncover single‐cell mitochondrial mutations and their patterns. However, it is important to note that our study serves as a proof‐of‐concept and has certain limitations. While the effect size estimates between mtDNA‐CN and age (*p* = 0.18) and sex (*p* = 0.14) were not statistically significant, they were in line with the anticipated trend of decreased mtDNA‐CN in older individuals and males (Yang et al., 2021). Although sex‐specific changes in the relationships linking lifespan, aging, and mitochondrial mutation rate are weak at the best (Cayuela et al., 2023; Junker et al., 2022), it is necessary to track the complete lifespan of a uniform group of male and female mice to obtain more reliable conclusions regarding changes in mtDNA contents or variations. Furthermore, as previously mentioned, our comparison of 23‐month‐old mouse tissues to those of 2‐month‐old mice may not only reflect aging‐related changes but also developmental influences. This is because 2‐month‐old mice are not yet fully mature. As a result, the observed patterns and current conclusions should be further explored and validated using a larger sample size that includes various aging stages, genders, and older adult mice (3–6 months old). Despite these limitations, we successfully developed and optimized the C4_mtscATAC‐seq as an alternative method to investigate the role of mtDNA variations and their interplay with the nuclear genome in the context of aging. Future studies can build upon our work to gain deeper insights into the intricate dynamics of mtDNA variations and their implications for the aging process.

## 4 METHODS

### 4.1 Sample preparation

We utilized human embryonic kidney epithelial cells (HEK‐293T) and the mouse preadipocyte cell line 3T3‐L1 obtained from ATCC (Manassas, VA). The cells were cultured in Dulbecco's Modified Eagle Medium (DMEM) supplemented with 10% Fetal Bovine Serum (FBS) and maintained in a humidified CO_2_ incubator at 37°C. Culturing conditions involved a 5% CO_2_ atmosphere. Prior to experimentation, the cells were cultured and harvested when they reached high density, approximately 90% confluency. HEK‐293T and 3T3‐L1 cells were cryopreserved in a cell storage solution consisting of 10% dimethyl sulfoxide (DMSO) and 90% FBS and stored in liquid nitrogen (−196°C). Two replicates were used for following library constructions.

The Institutional Animal Care and Use Committee of BGI reviewed and approved the animal experimental protocol. All procedures involving mice experiments in this study strictly adhered to ethical regulations and guidelines for Animal Experimentation of BGI, as well as the Guidelines for the Care and Use of Laboratory Animals in China (license number BGI‐IRB A21030‐T1). Two months old C57BL/6 male mice and 23 months aged C57BL/6 male mice were all obtained from Jiangsu Wukong Biotechnology Co., LTD (Nanjing, China) and were used for the study. B6.V‐Lep^Mice^ (ob/ob) and control mice were purchased from Beijing HFK Bioscience Co., Ltd (Beijing, China). Two technical replicates for each of two mice at each time point were used for libraries construction.

### 4.2 Single‐cell preparation

The spleen of two‐month‐old and 23‐month‐old C57BL/6J male mice were washed with cold PBS (Phosphate‐Buffered Saline). Subsequently, they were cut into small pieces using a blade and mashed using a plunger of a 1‐mL syringe. The pretreated spleen samples were then passed through a sterile cell filter with a pore size of 100 μm in a Petri dish containing 1 mL of cold DMEM (Dulbecco's Modified Eagle Medium) supplemented with 0.5% (w/v) BSA (Bovine Serum Albumin) and 2 mM EDTA (Ethylenediaminetetraacetic acid). The splenocytes were centrifuged, and the cell pellet was gently resuspend after removing the supernatant. The resuspend splenocytes were resuspended in 1× RBC (red blood cell) lysis buffer (ammonium chloride lysis buffer containing 0.1 mM EDTA, 155 mM NH_4_Cl, and 10 mM KHCO_3_ in MilliQ water). The splenocyte suspension was gently vortexed again and allowed to lyse the red blood cells at room temperature (RT) for 5 min. Next, 4 mL of PBS was added, and the splenocytes were centrifuged. After discarding the supernatant, the splenocytes were resuspended in 5 mL of PBS and passed through a sterile cell filter with a pore size of 30 μm in a Petri dish containing 1 mL of cold DMEM. The cell number was determined using a C‐chip counting chamber (VWR DHC‐N01). All centrifugations were performed at 500 g for 5 min at 4°C.

For the tibia and femora of 2‐month‐old and 23‐month‐old C57BL/6J mice, the bones were excised aseptically, and the marrow cavity was exposed as previously described (Zhao et al., 2022). The bone marrow and fragments of cancellous bone were collected. The bone marrow samples were transferred to sterile cell culture dishes and washed multiple times with cold PBS to harvest the marrow. The cell suspensions were then passed through a 70‐μm cellular filter (Falcon cell strainer, Franklin Lakes, NJ, USA). The cell suspensions were centrifuged at 125 g for 10 min at 4°C. The cell pellets were resuspended in PBS. To eliminate mature erythrocytes, the harvested bone marrow cells were treated with 1× RCB lysis buffer at room temperature for 5 min, followed by washing with PBS three times.

### 4.3 Library preparation and sequencing

For single‐cell ATAC sequencing (C4_scATAC‐seq), we followed a modified version of the single‐nucleus preparation workflow described previously (Mazid et al., 2022). Briefly, cells were lysed using 100 μL of lysis buffer A (10 mM Tris–HCl pH 7.5, 10 mM NaCl, 3 mM MgCl_2_, 0.1% NP40, 0.1% Tween‐20, 0.01% Digitonin, and 1% BSA) and then quenched with 1 mL of wash buffer (10 mM Tris–HCl pH 7.4, 10 mM NaCl, 3 mM MgCl_2_, and 1% BSA). The nuclear pellet was obtained by centrifugation at 400 g, 4°C, for 5 min, and washed twice with wash buffer through centrifugation at 400 *g*, 4°C, for 5 min. The nuclear pellet was then resuspended in PBS containing 1% BSA and stained with DAPI (4′,6‐diamidino‐2‐phenylindole) for counting the number of cell nuclei. The scATAC‐seq libraries were generated using the DNBelab C Series Single‐Cell ATAC Library Prep Set (MGI, #1000021878) and sequenced at MGI Tech Co., Ltd.

For single‐cell mitochondrial sequencing (C4_mtscATAC‐seq), cells were resuspended in 1 mL of precooled absolute methanol and incubated on ice for 10 min. After centrifugation at 4°C for 5 min at 300 *g*, the supernatant was discarded. The cell pellets were gently resuspended in 1 mL of precooled PBS and centrifuged again at 4°C for 5 min at 300 *g* to remove the supernatant. Resuspending the cell pellets in 100 μL of lysis buffer B (10 mM Tris–HCl pH 7.5, 10 mM NaCl, 3 mM MgCl2, 0.1% NP40, and 1% BSA), incubate on ice for 10 min, the cell membranes were gently perforated without complete dissolution and quenched with 1 mL of wash buffer (10 mM Tris–HCl pH 7.4, 10 mM NaCl, 3 mM MgCl_2_, and 1% BSA). The nuclear pellet was obtained by centrifugation at 400 *g*, 4°C, for 5 min, and washed twice with wash buffer through centrifugation at 400 *g*, 4°C, for 5 min. The nuclear pellet was then resuspended in PBS containing 1% BSA and stained with DAPI for counting the number of cell nuclei. The C4_mtscATAC‐seq libraries were generated using the DNBelab C Series Single‐Cell ATAC Library Prep Set (MGI, #1000021878) and sequenced at MGI Tech Co., Ltd. After the library construction, sequencing was carried out using the DNBSEQ sequencing platforms according to the manufacturer's instructions.

### 4.4 Quantitative PCR (qPCR)

We employed a previously described method (Quiros et al., 2017; Venegas & Halberg, 2012) for detecting mitochondrial DNA copy numbers in HEK‐293T and 3T3‐L1 cells. Briefly, cellular total DNA was extracted. Subsequently, mitochondrial DNA gene (mtDNA gene) and nuclear DNA gene (nuDNA gene) were separately amplified using specific primers (Table S6). The Ct values for the mtDNA gene and nuDNA gene were obtained. Finally, the mitochondrial copy number was calculated using the formula:

$$△\mathrm{Ct}=\mathrm{Ct}\;\left(\text{nuDNA gene}\right)-\mathrm{Ct}\;\left(\text{mtDNA gene}\right)$$

$$\text{Copies of mtDNA}=2\times 2^{△\mathrm{Ct}}$$

### 4.5 Data preprocessing and analysis

The reference genomes were download from the UCSC database. For human samples, the reference genome used was hg19, while for mouse samples, the reference genome used was mm10. All nuclear mtDNA segments regions of two reference genomes were masked. The raw sequencing data (Table S7) were then aligned to reference genomes by chromap (v0.2.3) (Zhang et al., 2021) (parameters “‐‐preset atac ‐‐bc‐error‐threshold 0 ‐‐trim‐adapters ‐x”). Only sequencing reads that aligned to the nuclear genome were retained for further analysis. The assignment of sequencing barcodes to cell barcodes was performed using d2c (v1.4.4, available at https://github.com/STOmics/d2c). With the alignment results for each cell, peak calling was conducted using macs2 (Zhang et al., 2008) with the parameters “‐B ‐q 0.001 –nomodel”. Subsequent analysis was carried out using the Signac v1.9.0 (Stuart et al., 2021) package in R v4.1.1. The following filtering criteria were applied to cells: (a) a transcription start site (TSS) enrichment score of at least 1.2, (b) a unique nuclear fragment count between 2000 and 50,000, and (c) a fraction of fragments in peaks (FRiP) higher than 0.2. Cells meeting these criteria were retained for further analysis.

To generate a set of shared peak regions, the resulting peaks from all samples in tissues or cell lines were merged, and a sparse count matrix was created based on these regions. Integration anchors across all samples were identified using Iterative Latent Semantic Indexing (LSI) projection for dimensionality reduction (Granja et al., 2019), and a single‐cell embedding was generated using t‐distributed stochastic neighbor embedding (TSNE) with dimensions ranging from 2 to 20. Further downstream analysis was conducted using default functions in Signac, including peak annotation, gene activity calculation, identification of genes associated with peaks, and transcription factor motif analysis. Cell type annotation was performed by using marker genes and an automated annotation process based on gene activity scoring. Initially, typical marker genes were used for annotation, and then integration with SingleR v1.8.1 (Ludwig et al., 2019) (ImmGenDat dataset), scMCA v0.2.0 (Sun et al., 2019), and scCATCH v3.2.1 (Shao et al., 2020) was performed to annotate clusters without marker genes.

### 4.6 mtDNA variation identification and filtering

For variation calling in whole genome sequencing data, we utilized GATK v4.1.8.1 (McKenna et al., 2010). For C4_mtscATAC‐seq data, we firstly employed mgatk (v0.6.2) (Lareau et al., 2021) with the parameter “‐kd” on position‐sorted BAM files extracted from mitochondrial genome mapping. Then we used the packages in signaC (https://stuartlab.org/signac/articles/mito) for jointing single‐cell mitochondrial DNA genotyping and DNA accessibility analysis, including dimension reduction and clustering, generate gene scores, visualize interesting gene activity scores, find informative mtDNA variants, compute the variant allele frequency for each cell, visualize the variants, and find differentially accessible peaks between clones. Specially, to filter the raw results, we utilized the “IdentifyVariants” package from Signac with the parameter “low_coverage_threshold = 10”. To ensure accurate identification of mtDNA variations, we implemented several quality control methods for filtering. Firstly, we aggregated the C4_mtscATAC‐seq profiles into pseudo‐bulk data and retained variants with at least a five‐fold difference in depth between the two alleles. Secondly, we filtered variants with >90% support on one strand at the bulk level. Thirdly, we filtered variants with a sequence depth lower than 250 at the bulk level. Fourthly, we set the minimum base quality of variants to 20. Fifthly, we applied a minimum mapping quality (MAPQ) threshold of 30. Sixthly, we required a maximum of two mismatches between the aligned reads and the reference genotype. Finally, we considered a minimum of 50 cells exhibiting a specific SNP as a requirement for it to be considered significant. In addition, we performed manual error correction to remove false positive result (m.310T>C) due to poly‐C structures in HEK‐293T samples. These steps were implemented to ensure the accuracy of mtDNA variations.

Furthermore, we utilized the ANNOVAR pipeline (v2020‐06‐07) (Wang et al., 2010) to annotate the functional impacts of mtDNA variations. We merged the cells from the same cell line or mice time point for all statistical analyses and plotting using the R language (version 4.1.1). Base R plotting and ggplot2 were utilized for figure generation. Statistical comparisons between two samples or cell types were performed using unpaired Wilcoxon rank sum tests.

## AUTHOR CONTRIBUTIONS

J.K., X.L., and D.W. conceived and leaded the study. F.L., J.K., X.S., C.W., Y.S., and L.J. carried out the experiments. F.L., Y.W., and C.Y. analyzed the data. X.L. and F.L. wrote the manuscript. J.K., X.S., L.J., and D.W. revised the manuscript. All authors read and approved the final manuscript.

## FUNDING INFORMATION

None.

## CONFLICT OF INTEREST STATEMENT

The authors declare no conflict of interests.

## Supporting information


Appendix S1.

Appendix S2.

## Data Availability

All raw sequencing data are available on China National GeneBank (CNP0004811) and Sequence Read Archive (PRJNA971679). All data supporting the findings of this study are included in the article and its Supplementary Data (Appendix S2). Codes for analysis C4_mtscATAC can be accessed from https://github.com/liufuyan2016/C4_mtscATAC.
