# Supplementary material for: Single‐cell mitochondrial sequencing reveals low‐frequency mitochondrial mutations in naturally aging mice
Source: Aging Cell. 2024 Jun 21;23(9):e14242. doi: 10.1111/acel.14242 (PMC11488324; doi:10.1111/acel.14242)
Supplement: Supplementary file 1 — Appendix S1. [file ACEL-23-e14242-s002.docx]

# SUPPLEMENTARY MATERIALS

## Single-Cell Mitochondrial Sequencing Reveals Low-Frequency Mitochondrial Mutations in Naturally Aging Mice

Fuyan Liu^1,*^, Xiaolin Sun^1,*^, Cai Wei^1,*^, Liu Ji^2,*^, Yali Song^1^, Chenlu Yang^1^,Yue Wang^1,3^, Xin Liu^1,4,#^, Daqing Wang^2,#^ and Jingmin Kang^1,4,#^

^1^ BGI Research, Beijing, China

^2^ Dalian Maternal and Child Health Hospital of Liaoning Province, Dalian, Liaoning, China

^3^ State Key Laboratory of Quality Research in Chinese Medicine and Institute of Chinese Medical Sciences, University of Macau, Macao, China

^4^ BGI Research, Shenzhen, China

^*^ These authors contributed equally.

^#^ Correspondence

## SUPPLEMENTARY FIGURES


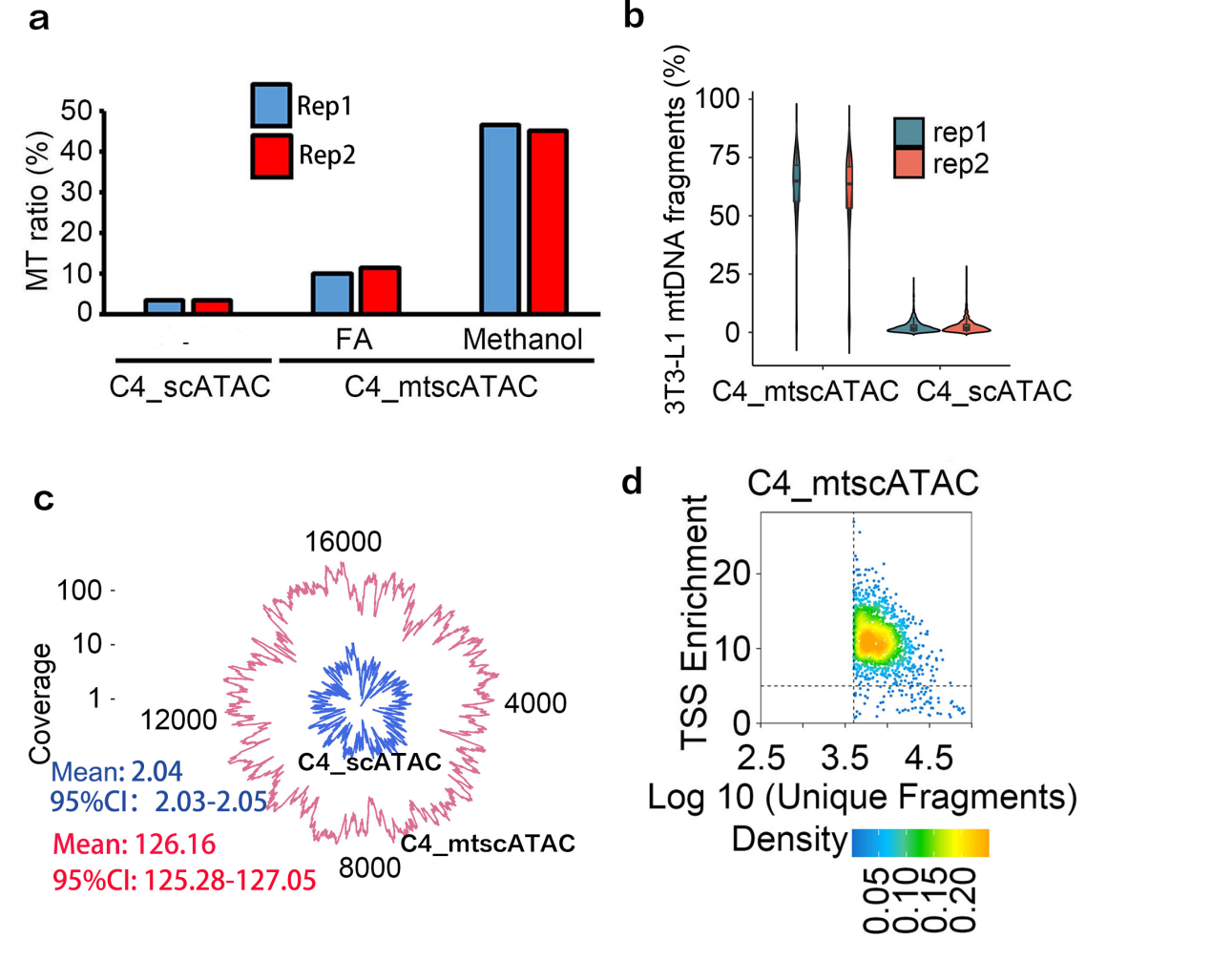


**Figure S1. C4_mtscATAC-seq of the mouse 3T3-L1 cell line.**

**a.** Percentages of mitochondria content in 3T3-L1 cell lines for screened C4_scATAC and C4_mtscATAC. Different fixation agents, formaldehyde (FA), and methanol were tested in C4_mtscATAC. Each experiment included two biological replicates.**b.** Percentage results of mtDNA fragments in all the sequenced fragments per single cell in C4_scATAC-seq and C4_mtscATAC-seq of 3T3-L1 cells.**c.** Improvement in overall mitochondrial genome coverage by C4_mtscATAC compared to the original protocol C4_scATAC in the 3T3-L1 cell line.**d.** Quality control filters for C4_mtscATAC data in mouse (3T3-L1 cell line). The scatter plot shows the number of unique ATAC-seq nuclear fragments in each individual cell, represented by dots, along with the Transcription Start Site (TSS) enrichment of all fragments within that cell. In panels b and c, each condition represents the top 1,000 cells (based on chromatin complexity) for each replicate.


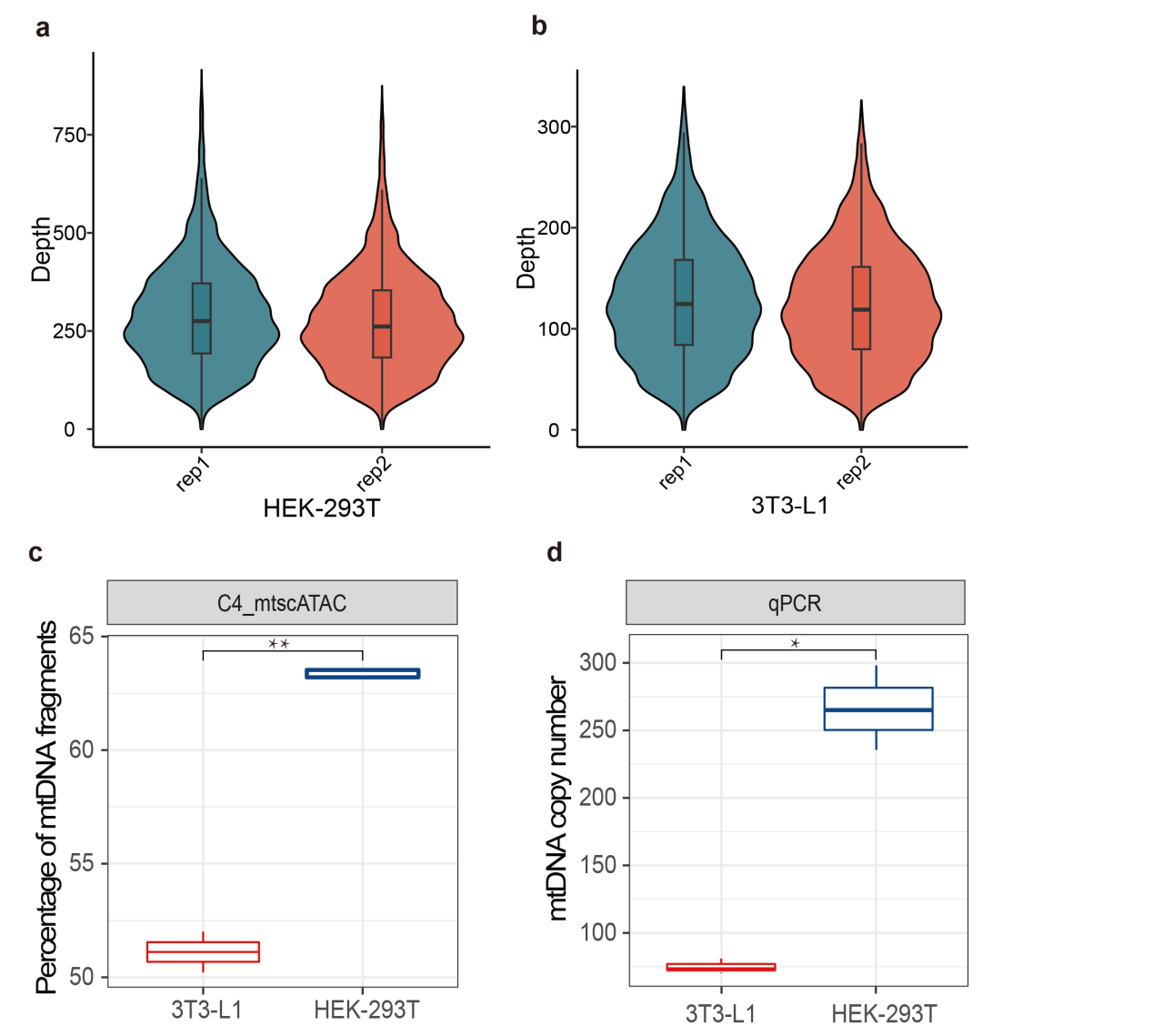


**Figure S2. The mitochondrial sequencing depth and copy number of two cell lines.**

**a**. distribution plot of mitochondrial sequencing depth for each cell in the HEK-293T cell line.**b**. distribution plot of mitochondrial sequencing depth for each cell in the 3T3-L1 cell line.**c**. Compare the percentage of mtDNA fragment distributions between 3T3-L1 and HEK-293T cell lines from C4_mtscATAC experiments.**d**. Compare the mtDNA copy number between 3T3-L1 and HEK-293T cell lines from three replicates qPCR experiments.Statistical significance was determined using the Wilcoxon test. * denotes p-values between 0.01 and 0.05, ** denotes p-values between 0.001 and 0.01.


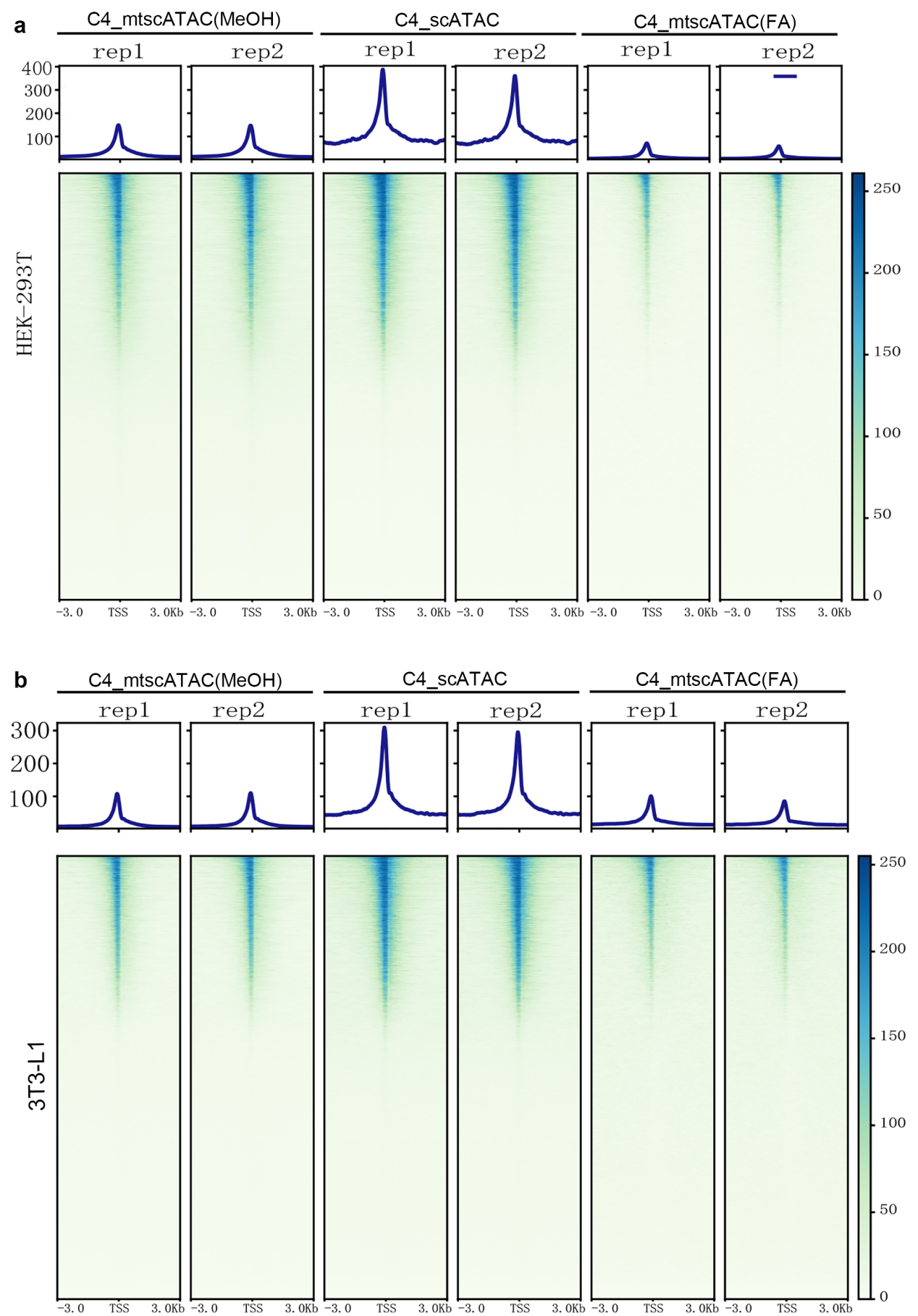


**Figure S3. Profile heatmap around the Transcription Start Site (TSS) of RefSeq genes.**

The heatmaps illustrate the normalized ATAC read density distributions of Methanol (MeOH)-fixed C4_mtscATAC C4_scATAC, and formaldehyde (FA)-fixed C4_mtscATAC within the TSS region (±3 kb). Blue color indicates the enrichment of ATAC signals at the TSS. The top enrichment plot represents the normalized density of mtC4_scATAC(MeOH), C4_scATAC, and C4_mtscATAC(FA) samples across the TSS region (±3 kb).**a.** Heatmap and enrichment plot for HEK-293T cells.**b.** Heatmap and enrichment plot for 3T3-L1 cells.


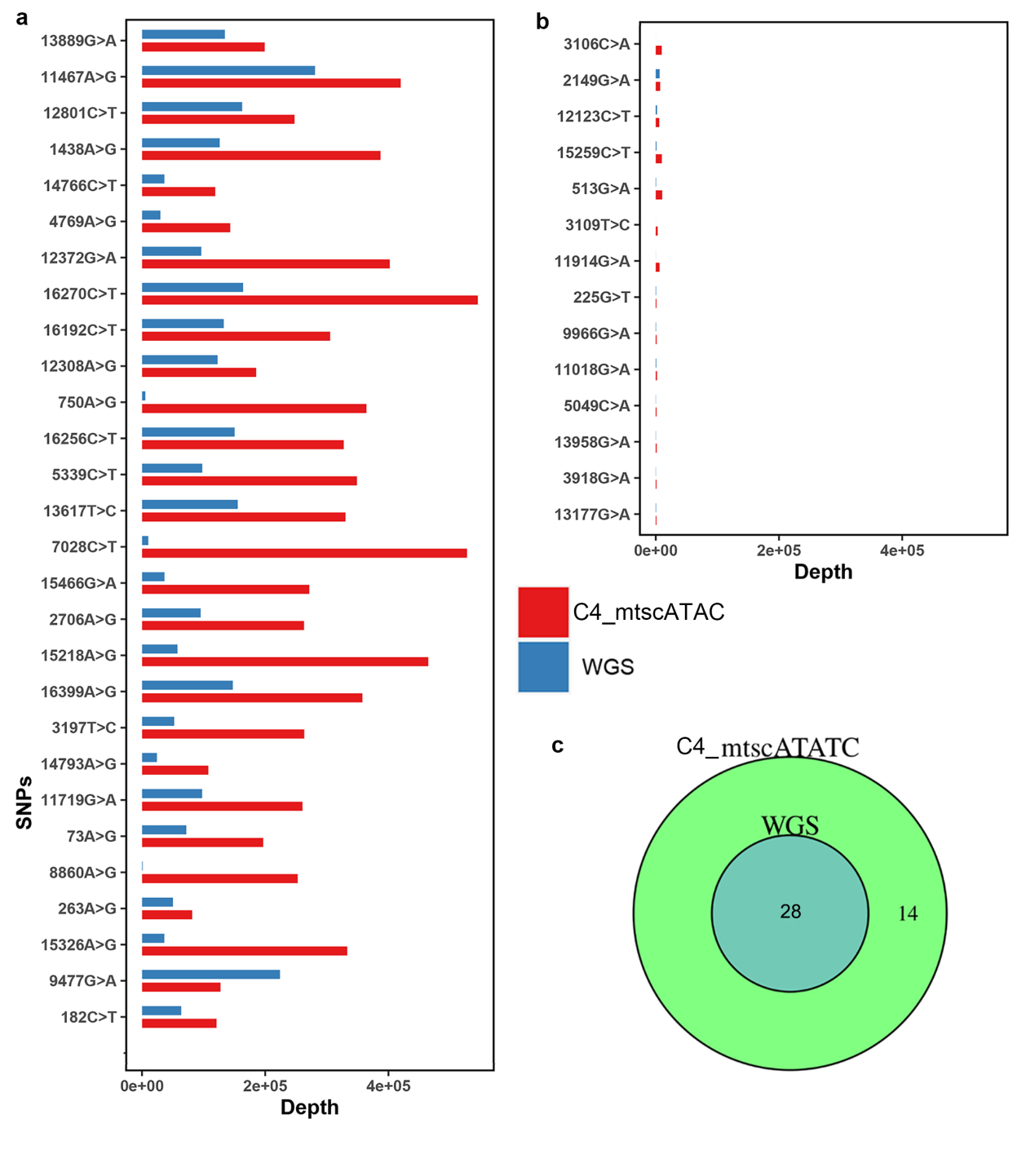


**Figure S4. Single-cell mtDNA variations in HEK-293T cell line. a,b,** The bar plot illustrates the variation depth in HEK-293T cells, along with the number of overlapping mutations identified by Whole Genome Sequencing (WGS) and C4_mtscATAC. The blue bar represents WGS data, and the red bar represents the aggregated C4_mtscATAC data. **c,** The Venn diagram, positioned in the center of the bar plot, illustrates the count of overlapping mutations between WGS and C4_mtscATAC.


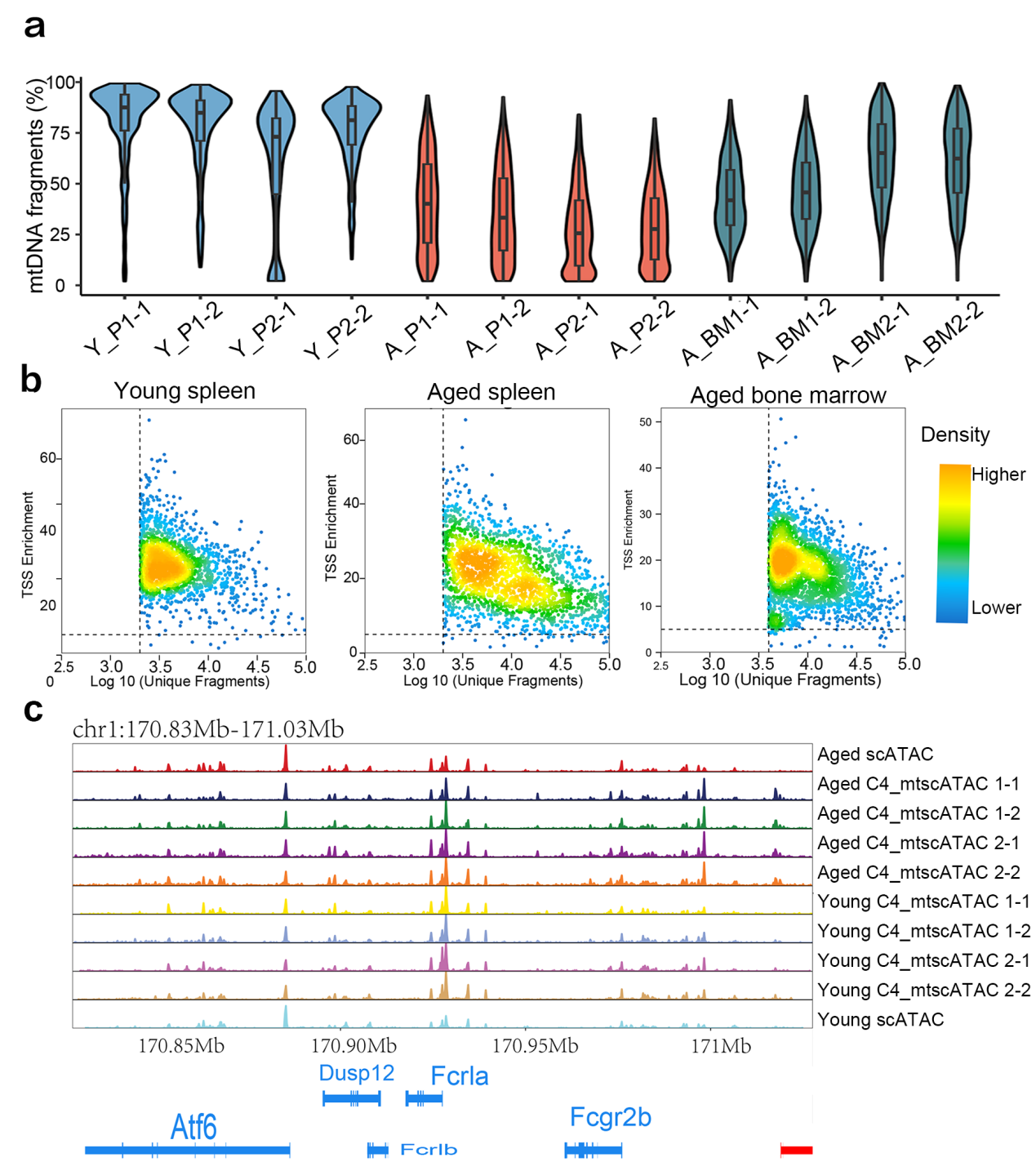


**Figure S5. Quality control of C4_mtscATAC-seq data in mouse tissues.**

**a.** Violin plots displaying the distribution of mitochondrial content per cell in the young spleen (Y_P1-1-Y_P2-2), aged spleen (A_P1-1-A_P2-2), and aged bone marrow (A_BM1-1-A_BM_2-2) samples.**b.** Scatter plot illustrating the number of unique ATAC-seq nuclear fragments in each individual cell, represented by dots, along with the TSS enrichment of all fragments within that cell. **c.** Accessible chromatin landscapes aggregated from single cells located in the chr1:170.83Mb-171.03Mb region were analyzed using both scATAC-seq and C4_mtscATAC-seq in the young and aged spleens.


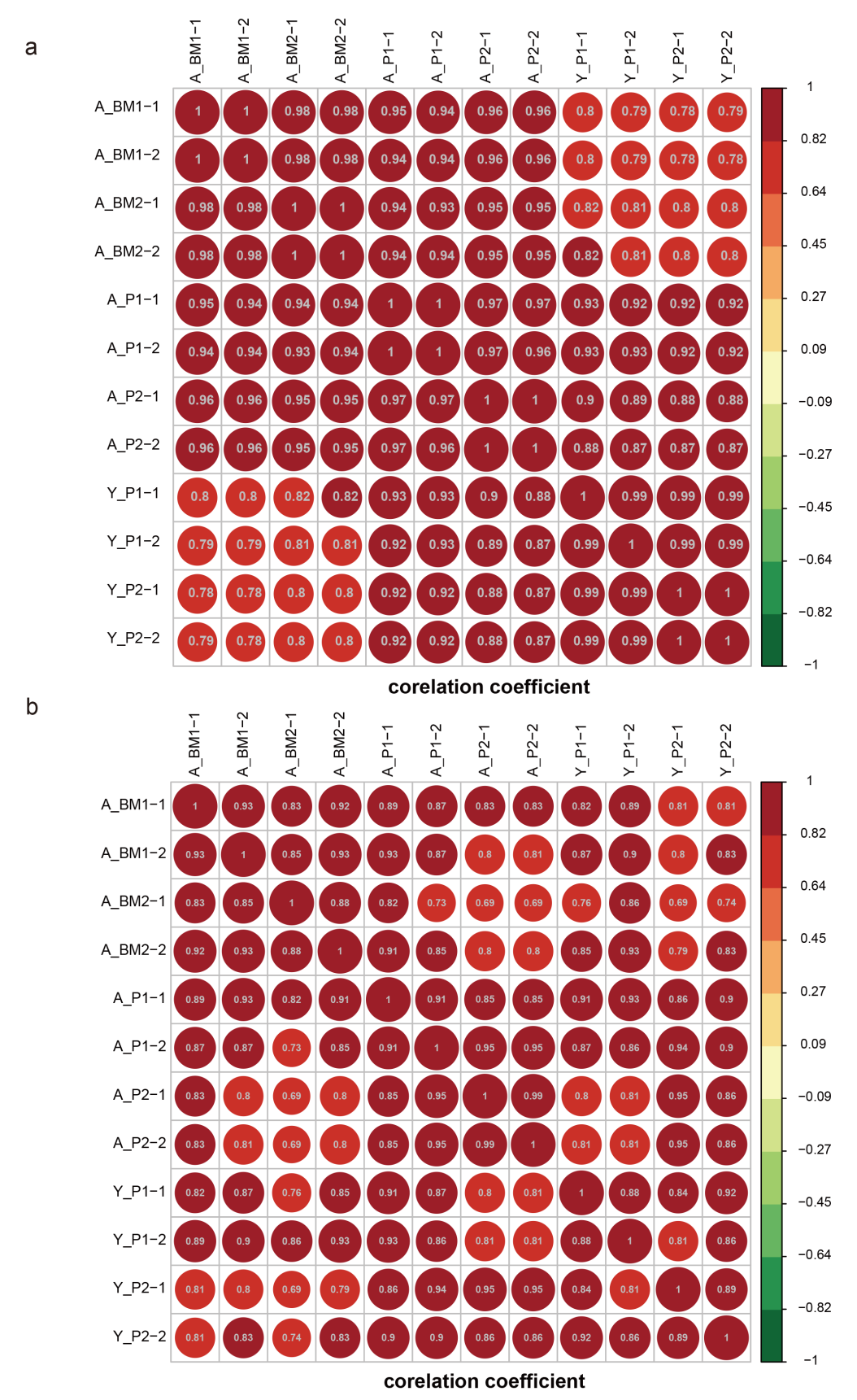


**Figure S6. Pearson correlation heatmap for chromatin accessibility and mitochondrial coverage in mouse tissues.**

**a.** Pearson correlation heatmap depicting the pairwise sample correlation based on common chromatin accessibility peaks of C4_mtscATAC-seq data across all samples.**b**.Pearson correlation heatmap depicting the pairwise sample correlation based on mitochondrial sequencing depth of C4_mtscATAC-seq data across all samples.


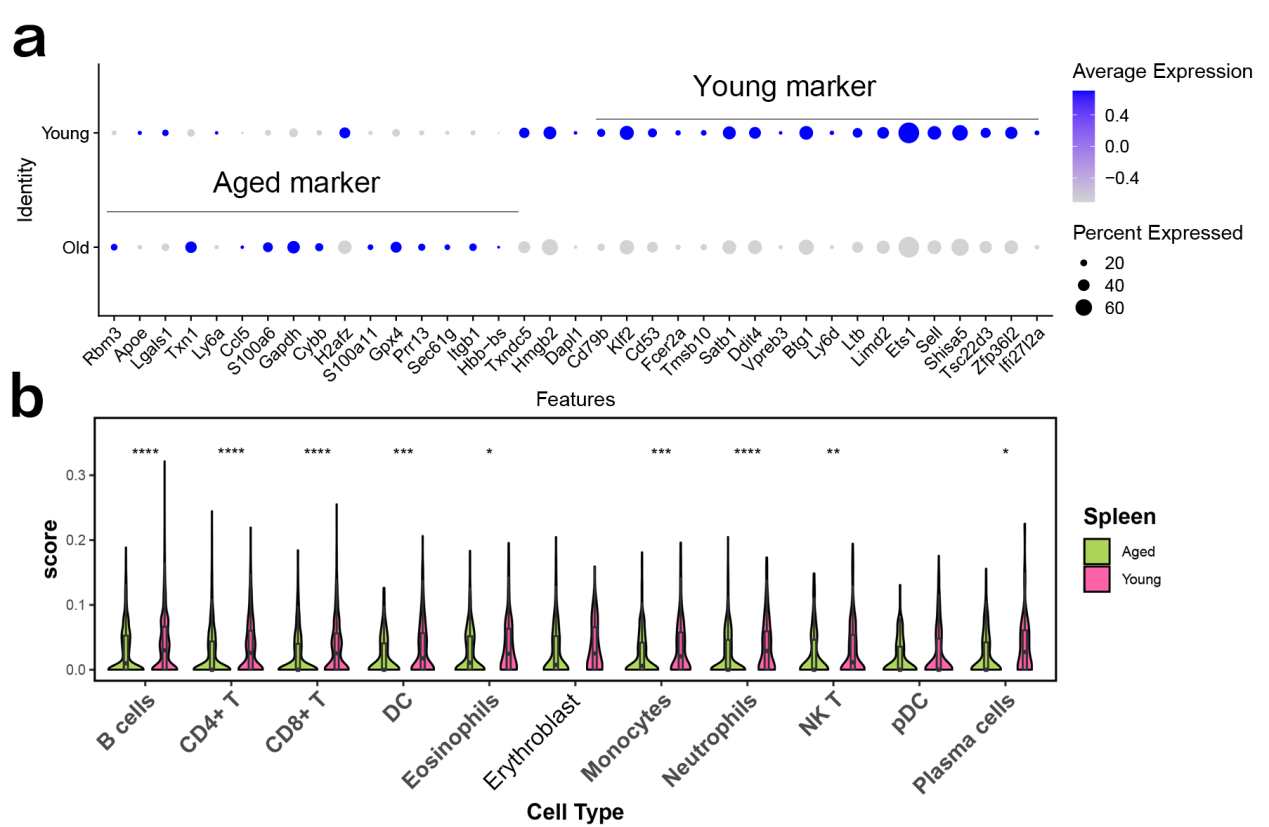


**Figure S7. Validation of differences in mitochondrial content through marker genes.**

**a.** Dot plot illustrating the average expression scores of genes associated with the coefficient of spleen aging that exhibit differential expression between young and aged samples. The size of each dot represents the proportion of cells expressing the respective genes.**b.** Comparison of gene activity scores associated with mtDNA replication and transcription between young and aged spleen tissues in mice. The mtDNA replication genes include *Polg*, *Polg2*, *Mgme1*, *Primpol*, *Rnaseh1*, *Ssbp1*, *Twnk*, and *Dna2*. The mtDNA transcription genes include *Tfam*, *Tfb2m*, *Tefm*, and *Polrmt*. Statistical significance was determined using the Wilcoxon test. * denotes p-values between 0.01 and 0.05, ** denotes p-values between 0.001 and 0.01, *** denotes p-values between 0.0001 and 0.001, and **** denotes p-values less than 0.0001**.**


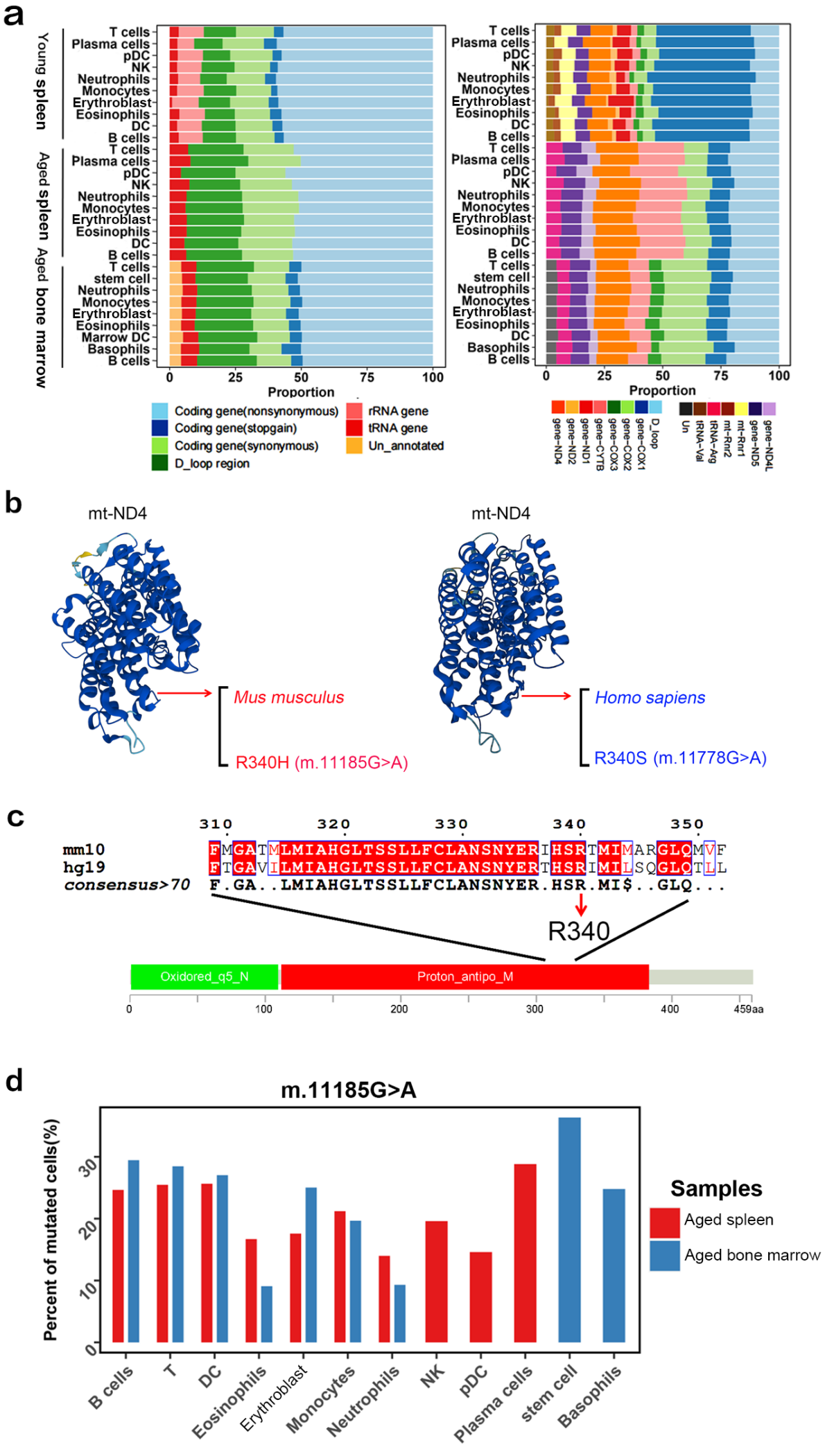


**Figure S8. Functional annotation of mtDNA variations.**

**a.** The left panel illustrates the percentages of cells from young and aged mice tissues with mtDNA variations annotated in proteins (nonsynonymous and synonymous mutations), D-loop, rRNA, and tRNA. The right panel shows the percentages of cells from young and aged mice tissues with mtDNA variations annotated in different mitochondrial respiratory chain subunits such as *ND1*, *ND2*, *ND3*, *ND4*, etc.**b.** The predicted structure of *ND4*, analyzed using the online tool AlphaFold (https://www.alphafold.ebi.ac.uk/), shows that human and mouse mtDNA variations are located in the same position of *ND4*, suggesting that the m.11185G>A mutation in mice may be a pathogenic mtDNA variation causing diseases.**c.** The domain of *ND4* was analyzed using the online analysis tool cBioPortal (http://www.cbioportal.org/), indicating that the amino acids encoded by the m.11185G>A mutation are within the functional domain of *ND4*.**d.** The distribution of cells with positive m.11185G>A mutation in individual clusters is demonstrated.

## SUPPLEMENTARY TABLES

**Table S1. The quality assessment C4_mtscATAC and C4_scATAC libraries of the mix cell lines.**

| **Sample name** | **Species** | **Estimated number of cells** | **Median fragments per cell** | **Median fraction of fragments overlapping TSSs** | **Median fraction of fragments overlapping peaks** |
| --- | --- | --- | --- | --- | --- |
| **mtscATAC_FA rep1** | hg19 | 625 | 636 | 21.18% | 5.41% |
|  | mm10 | 3,847 | 682 | 10.14% | 5.52% |
| **mtscATAC_FA rep2** | hg19 | 485 | 657 | 19.25% | 3.54% |
|  | mm10 | 4,284 | 725 | 8.31% | 3.64% |
| **mtscATAC Methenol_rep1** | hg19 | 2,537 | 11,044 | 19.77% | 25.69% |
|  | mm10 | 1,708 | 7,366 | 20.39% | 34.44% |
| **mtscATAC Methenol_rep2** | hg19 | 2,565 | 10,546 | 20% | 25.59% |
|  | mm10 | 1,869 | 7,088 | 20.13% | 33.26% |
| **scATAC rep1** | hg19 | 4,572 | 7,468 | 36.12% | 57.84% |
|  | mm10 | 4,212 | 7,744 | 33.34% | 69.17% |
| **scATAC rep2** | hg19 | 4,382 | 7,235 | 35.68% | 56.82% |
|  | mm10 | 4,069 | 7,627 | 33.04% | 68.70% |

**Table S2. The quality evaluation of the C4_mtscATAC libraries from mouse spleen and bone marrow tissues.**

| **Sample** | **Estimated number of cells** | **Median fragments per cell** | **Median fraction of fragments overlapping TSSs** | **Median fraction of fragments overlapping peaks** |
| --- | --- | --- | --- | --- |
| **Young spleen1-1** | 2,437 | 3,046 | 37.07 | 52.36 |
| **Young spleen1-2** | 2,630 | 2,858 | 37.75 | 53.33 |
| **Young spleen2-1** | 3,102 | 4,552 | 36.91 | 55.31 |
| **Young spleen2-2** | 2,533 | 4,679 | 37.58 | 55.67 |
| **Aged spleen1-1** | 2,477 | 7,067 | 29.73 | 41.68 |
| **Aged spleen1-2** | 3,734 | 6,948 | 26.87 | 37.96 |
| **Aged spleen2-1** | 2,785 | 13,969 | 19.84 | 25.26 |
| **Aged spleen2-2** | 2,658 | 14,643 | 19.22 | 24.97 |
| **Aged bone marrow1-1** | 4,408 | 4,852 | 21.44 | 27.68 |
| **Aged bone marrow1-2** | 2,951 | 7,362 | 20.34 | 26.64 |
| **Aged bone marrow2-1** | 2,047 | 4,822 | 22.49 | 27.55 |
| **Aged bone marrow2-2** | 4,682 | 2,155 | 21.51 | 26.93 |

**Table S3. The number of cells identified in each tissue.**

**A.****The number of cells in spleens.**

| **Cell Type** | **Young spleen** | **Aged spleen** |
| --- | --- | --- |
| **B cells** | 3,792 | 2,581 |
| **CD4+ T** | 1,664 | 1,155 |
| **CD8+ T** | 1,099 | 726 |
| **DC** | 555 | 195 |
| **Eosinophils** | 133 | 719 |
| **Erythroblast** | 68 | 381 |
| **Monocytes** | 326 | 547 |
| **Neutrophils** | 186 | 1,001 |
| **NK T** | 520 | 199 |
| **pDC** | 251 | 137 |
| **Plasma cells** | 75 | 288 |

**B.The number of cells in bone marrows.**

| **Cell Type** | **Aged bone marrow** |
| --- | --- |
| **B cells** | 842 |
| **Basophils** | 121 |
| **DC** | 185 |
| **Eosinophils** | 1,145 |
| **Erythroblast** | 979 |
| **Monocytes** | 1,129 |
| **Neutrophils** | 3,280 |
| **stem cell** | 410 |
| **T cells** | 506 |

**Table S4. Cell type annotation of mouse spleen and bone marrow tissues.**

| **Tissue** | **Cluster** | **Cell Type** | **Marker gene** |
| --- | --- | --- | --- |
|  |  |  |  |
| **Spleen** | 0,2,5 | B cells | Pax5,Cd79a,Blnk,Cd19,Ms4a1 |
| **Spleen** | 1 | CD4+ T | Bcl11b,Cd4,Cd3d,Nkg7 |
| **Spleen** | 3 | CD8+ T | Bcl11b,Cd8a,Cd3d,Nkg7 |
| **Spleen** | 4,13 | Neutrophils | S100a8,Ltf,Lyz2,Csf3r |
| **Spleen** | 6 | Monocytes | Csf1r,Ly6c2,Nr4a1 |
| **Spleen** | 7 | Eosinophils | Prg2 |
| **Spleen** | 8 | DC | Cd209a,Itgax |
| **Spleen** | 9 | NK | Gzma,Ncr1,Nkg7,Mcpt8 |
| **Spleen** | 10 | Erythroblast | Gypa,Gata1 |
| **Spleen** | 11 | pDC | NA |
| **Spleen** | 12 | Plasma cells | Sdc1,Jchain |
| **Bone marrow** | 0,1 | Neutrophils | S100a8,Ltf,Lyz2 |
| **Bone marrow** | 2 | Monocytes | Csf1r,Ly6c2,Nr4a1 |
| **Bone marrow** | 3,5 | Erythroblast | Gypa,Gata1 |
| **Bone marrow** | 4 | Eosinophils | Prg2 |
| **Bone marrow** | 6 | stem cells | NA |
| **Bone marrow** | 7,8 | B cells | Pax5,Cd79a,Blnk,Cd19,Ms4a1 |
| **Bone marrow** | 9 | T cells | Bcl11b,Nkg7 |
| **Bone marrow** | 10 | DC | NA |

**Table S5. Score values of mitochondria-related genes signature in the spleen.**

| **Spleen** | **Aged** | **Young** | **Fold（Young/Aged)** |
| --- | --- | --- | --- |
| **B cells** | 0.029 | 0.0389 | 1.34 |
| **CD4+ T** | 0.0252 | 0.036 | 1.43 |
| **CD8+ T** | 0.0231 | 0.0344 | 1.49 |
| **DC** | 0.0211 | 0.0331 | 1.57 |
| **Eosinophils** | 0.0278 | 0.0355 | 1.28 |
| **Erythroblast** | 0.0286 | 0.0353 | 1.23 |
| **Monocytes** | 0.024 | 0.0345 | 1.44 |
| **NK** | 0.0229 | 0.0312 | 1.36 |
| **Neutrophils** | 0.0249 | 0.0373 | 1.50 |
| **Plasma cells** | 0.024 | 0.0354 | 1.48 |
| **pDC** | 0.019 | 0.026 | 1.37 |

**Table S6. qPCR results of mitochondrial DNA copy number in HEK-293T and 3T3-L1**

| **Gene** | **Primer name** | **Primer sequence** | **CT 1** | **CT 2** | **CT 3** | **Average** | **△CT** | **Copies** |
| --- | --- | --- | --- | --- | --- | --- | --- | --- |
| **mtDNA-tRNA (Leu-UUR)** | **mtDNA-tRNA F3212** | CACCCAAGAACAGGGTTTGT | 16.27 | 16.30 | 16.28 | 16.28 | 7.05 | 265.03 |
|  | **mtDNA-tRNA R3319** | TGGCCATGGGTATGTTGTTA |  |  |  |  |  |  |
| **nuDNA β2-microglobulin** | **nuDNA-ß2M F594** | TGCTGTCTCCATGTTTGATGTATCT | 23.32 | 23.18 | 23.50 | 23.33 |  |  |
|  | **nuDNA-ß2M R679** | TCTCTGCTCCCCACCTCTAAGT |  |  |  |  |  |  |
| **mtDNA ND1** | **mtDNA-ND1 forward** | CTAGCAGAAACAAACCGGGC | 14.08 | 14.04 | 13.89 | 14.00 | 5.23 | 75.60 |
|  | **mtDNA-ND1 reverse** | CCGGCTGCGTATTCTACGTT |  |  |  |  |  |  |
| **nuDNA HK2** | **nuDNA-HK2 forward** | GCCAGCCTCTCCTGATTTTAGTGT | 19.25 | 19.23 | 19.23 | 19.24 |  |  |
|  | **nuDNA-HK2 reverse** | GGGAACACAAAAGACCTCTTCTGG |  |  |  |  |  |  |

**Table S7. The single cell sequencing data generated from this study.**

| **Sample name** | **BaseSum(bp)** | **Q30** | **Mapping** | **SRA** |
| --- | --- | --- | --- | --- |
| **scATAC rep1** | 48,777,425,760 | 92.60 | 78.1 | SRR24601276 |
| **scATAC rep2** | 46,810,585,800 | 92.13 | 78.51 | SRR24601275 |
| **mtscATAC_FA rep1** | 55,905,090,360 | 93.65 | 65.42 | SRR24601274 |
| **mtscATAC FA rep2** | 49,581,349,080 | 93.55 | 66.12 | SRR24601273 |
| **mtscATAC Methenol_rep1** | 57,619,395,720 | 94.36 | 87.59 | SRR24601272 |
| **mtscATAC Methenol_rep2** | 57,426,249,240 | 94.38 | 86.58 | SRR24601271 |
| **Young spleen1-1** | 63,087,536,370 | 93.30 | 88.60 | SRR24518043 |
| **Young spleen1-2** | 48,311,179,560 | 92.80 | 87.39 | SRR24518042 |
| **Young spleen2-1** | 49,575,517,560 | 92.57 | 88.89 | SRR24518040 |
| **Young spleen2-2** | 61,077,240,600 | 93.32 | 89.89 | SRR24518037 |
| **Aged spleen1-1** | 59,931,533,760 | 95.44 | 89.97 | SRR24518041 |
| **Aged spleen1-2** | 58,970,349,000 | 95.11 | 90.28 | SRR24518036 |
| **Aged spleen2-1** | 58,550,560,680 | 95.046 | 90.60 | SRR24518035 |
| **Aged spleen2-2** | 55,903,622,760 | 94.498 | 90.11 | SRR24518033 |
| **Aged bone marrow1-1** | 57,503,331,960 | 95.71 | 90.60 | SRR24518032 |
| **Aged bone marrow1-2** | 56,569,974,240 | 95.83 | 90.19 | SRR24518034 |
| **Aged bone marrow2-1** | 53,526,091,080 | 95.15 | 89.66 | SRR24518039 |
| **Aged bone marrow2-2** | 55,122,408,240 | 95.60 | 90.08 | SRR24518038 |

**SUPPLEMENTARY DATA**

SD01. HEK-293T mtSNP identification.

SD02. HEK-293T mtSNP annotation.

SD03. mtSNP identification in mouse spleen and bone marrow tissues.

SD04. mtSNP annotation in mouse spleen and bone marrow tissues.

SD05. Differential peaks of top three mtSNPs in aging spleen tissues.

SD06. Differential peaks of top three mtSNPs in aging bone marrow tissues.
